# Cyclic Polyesters with Closed‐Loop Recyclability from A New Chemically Reversible Alternating Copolymerization

**DOI:** 10.1002/advs.202306072

**Published:** 2023-11-30

**Authors:** Xiaoxian Lu, Xun Zhang, Chengjian Zhang, Xinghong Zhang

**Affiliations:** ^1^ National Key Laboratory of Biobased Transportation Fuel Technology International Research Center for X Polymers Department of Polymer Science and Engineering Zhejiang University Hangzhou 310027 China

**Keywords:** alternating copolymerization, cyclic polymers, polyesters, recyclable polymers, sustainable polymers

## Abstract

Polyesters with both cyclic topology and chemical recyclability are attractive. Here, the alternating copolymerization of cyclic anhydride and *o*‐phthalaldehyde to synthesize a series of cyclic and recyclable polyesters are reported for the first time. Besides readily available monomers, the copolymerization is carried out at 25 °C, uses common Lewis/Brønsted acids as catalysts, and achieves high yields within 1 h. The resulting polyesters possess well‐defined alternating sequences, high‐purity cyclic topology, and tunable structures using distinct two monomer sets. Of interest, the copolymerization manifests obvious chemical reversibility as revealed by kinetic and thermodynamic studies, making the unprecedented polyesters easy to recycle to their distinct two monomers in a closed loop at high temperatures. This work furnishes a facile and efficient method to synthesize cyclic polyesters with closed‐loop recyclability.

## Graphical abstract

The cationic copolymerization of cyclic anhydride and *o*‐phthalaldehyde demonstrates alternating and chemical reversible characteristics, yielding novel polyesters with alternating sequence, high‐fidelity cyclic topology, closed‐loop chemical recyclability, and easy‐to‐tune structure.

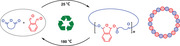

## 1 Introduction

Plastics are almost everywhere. The annual global output of plastics exceeds 380 million tons nowadays.^[^ ^1^, ^2^ ^]^ The plastics we discard are rarely recycled or incinerated in waste‐to‐energy facilities, while most of them are relegated to landfills or leaked into our environment, leading to a waste of resources and environmental pollution.^[^ ^3^, ^4^ ^]^ The evolution of chemically recyclable polymers that can be effectively recycled into their starting monomers furnishes a promising solution to address the challenge of plastic sustainability.^[^ ^5^, ^6^, ^7^, ^8^, ^9^, ^10^, ^11^, ^12^, ^13^, ^14^, ^15^, ^16^, ^17^ ^]^ The strategy can enable the circular use of resources and fundamentally solve the problem of polymer waste disposal. One of the hot topics in current polymer science is to develop chemically recyclable polymers.

**Figure 1:**
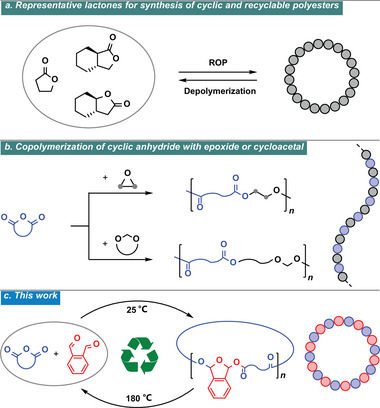
Polyester synthesis. a) ROP of representative lactones for the synthesis of cyclic and recyclable polyester. b) Alternating copolymerization of cyclic anhydride and epoxide/cycloacetal to yield linear polyesters. c) This work: copolymerization of cyclic anhydrides and OPA to prepare alternating and cyclic polyesters with closed‐loop chemical recyclability.

Ring‐opening polymerization (ROP) of low‐strain lactones has been well demonstrated as a versatile method for the preparation of recyclable polyesters.^[^ ^18^, ^19^, ^20^, ^21^, ^22^, ^23^, ^24^, ^25^ ^]^ Interestingly, a few lactones can form polyesters with cyclic topology through state‐of‐the‐art catalysis, such as γ‐butyrolactone and six‐five bicyclic lactones reported by Chen and co‐workers (**Figure** **1**a).^[^ ^26^, ^27^, ^28^ ^]^ The synthesis of cyclic polymers of sufficient quantity and purity remains a formidable challenge.^[^ ^29^, ^30^, ^31^, ^32^, ^33^, ^34^, ^35^, ^36^, ^37^ ^]^ Cyclic polymers are a captivating class of polymers because of their lack of chain ends. The special architecture combined with the steric constraint enables cyclic polymers with distinctive physicochemical properties.^[^ ^38^, ^39^, ^40^ ^]^

The alternating copolymerization of cyclic anhydride with epoxide or cycloacetal is another versatile strategy for polyester synthesis (Figure 1b).^[^ ^41^, ^42^, ^43^, ^44^, ^45^, ^46^, ^47^, ^48^, ^49^ ^]^ Cyclic anhydrides are sustainable monomers for polymer synthesis, which can be industrially produced from the intramolecular dehydration of biorenewable and low‐cost dicarboxylic acids.^[^ ^50^ ^]^ The resulting polyesters usually possess the linear topology sometimes mixed with a little cyclic topology generated from the back‐biting side reaction.^[^ ^51^, ^52^ ^]^ Also, due to the near irreversibility of the copolymerization, such polyesters can hardly be directly recycled back to original monomers.

Here, we demonstrate the alternating copolymerization of cyclic anhydride and *o*‐phthalaldehyde (OPA) with a cationic mechanism (Figure 1c). OPA is a commonly used chemical disinfectant for dental and medical instruments and is also used as an intermediate in pharmaceutical synthesis.^[^ ^53^ ^]^ At 25 °C, using the two distinct monomer sets, our method yields various novel polyesters with a high purity of cyclic topology as well as a complete alternating sequence. Interestingly, owing to the evident chemical reversible feature of the copolymerization, the resulting cyclic polyesters can be directly depolymerized into the two distinct monomers at high temperatures. We recently reported the alternating copolymerization of cyclic anhydride and aldehyde to yield chemically recyclable polymers with linear topology.^[^ ^54^ ^]^ Compared with that study, this work provides a series of polyesters with high‐purity cyclic topology.

## 2 Results and Discussion

We carried out the copolymerization of OPA and glutaric anhydride (GA) with the cationic mechanism. We are grateful that several common Lewis/Brønsted acids are effective catalysts for copolymerization under mild conditions. At 25 °C, for 1 h, with the feeding ratio of [OPA]_0_:[GA]_0_:[catalyst] = 100:100:1, using CH_2_Cl_2_ as a solvent, the catalysts of BF_3_·Et_2_O, InCl_3_, InBr_3_, NH(OTf)_2_, TfOH, and H_2_SO_4_ achieved monomer conversions of 80 – 86%, yielding the polyester of poly(OPA‐*alt*‐GA) with number‐average molecular weights (*M*_n_) of 9.7 – 13.2 kDa and polydispersities (*Ð*) of 1.3 – 1.5 (entries 1–6, **Table** **1**). We then synthesized the polymer with higher *M*_n_ by reducing the amount of catalyst. With the feeding ratio of [OPA]_0_:[GA]_0_:[catalyst] = 1000:1000:1, at 25 °C for 1 h, the catalysts yielded the copolymer with high *M*_n_ of 19.0–32.0 kDa, while monomer conversions were in the range of 82 – 86%, suggesting an efficient manner. The double C═O bonds of OPA were transformed into the acetal bond, which was similar to the phenomenon that occurred in the OPA homopolymerization. The resulting polyesters possess the complete OPA‐GA alternating sequence without any OPA‐ or GA‐homopolymer units observed from the NMR analysis (Figure S1, Supporting Information). For comparison, with the anionic/coordination mechanism and under the same reaction conditions, the OPA and GA copolymerization did not occur using the catalyst systems of triethyl borane/bis(triphenylphosphine)iminium chloride (PPNCl)^[^ ^55^ ^]^ and (Salen)CrCl/PPNCl,^[^ ^41^ ^]^ which were widely applied in epoxide and anhydride copolymerization. As a result, the alternating copolymerization of OPA and GA was first achieved by the cationic mechanism.

**Table 1:** Copolymerization of OPA (Br‐OPA) with various cyclic anhydrides.

entry^a^ ^)^	Cat.	M1	M2	[M1]:[M2]:[Cat.]	Conv.[%]^b^ ^)^	*M*_n [_kDa]^c^ ^)^	*Ð* ^c^ ^)^
1	BF_3_·Et_2_O	OPA	GA	100:100:1	85	13.2	1.4
2	InCl_3_	OPA	GA	100:100:1	84	10.5	1.3
3	InBr_3_	OPA	GA	100:100:1	84	9.7	1.4
4	NH(OTf)_2_	OPA	GA	100:100:1	80	11.2	1.5
5	TfOH	OPA	GA	100:100:1	86	10.6	1.4
6	H_2_SO_4_	OPA	GA	100:100:1	83	11.2	1.4
7	BF_3_·Et_2_O	OPA	GA	1000:1000:1	82	27.6	1.5
8	InCl_3_	OPA	GA	1000:1000:1	83	20.6	1.5
9	InBr_3_	OPA	GA	1000:1000:1	86	32.0	1.4
10	NH(OTf)_2_	OPA	GA	1000:1000:1	85	19.0	1.9
11	BF_3_·Et_2_O	OPA	–	100:100:1	0	–	–
12	BF_3_·Et_2_O	–	GA	100:100:1	0	–	–
13	BF_3_·Et_2_O	OPA	MGA	100:100:1	85	6.3	1.4
14	BF_3_·Et_2_O	OPA	DMGA	100:100:1	59	4.5	1.5
15	BF_3_·Et_2_O	OPA	*i*BuGA	100:100:1	65	4.4	1.2
16	BF_3_·Et_2_O	OPA	TDGA	100:100:1	82	6.3	1.4
17	BF_3_·Et_2_O	Br‐OPA	GA	100:100:1	40	5.6	1.2

^a)^ The copolymerization was performed at 25 °C, for 1 h, in CH_2_Cl_2_, [OPA]_0_ = 3.06 m;

^b)^ Conversion of cyclic anhydrides, determined by ^1^H NMR spectroscopy;

^c)^ Determined by GPC in THF, calibrated with polystyrene standards.

**Figure 2:**
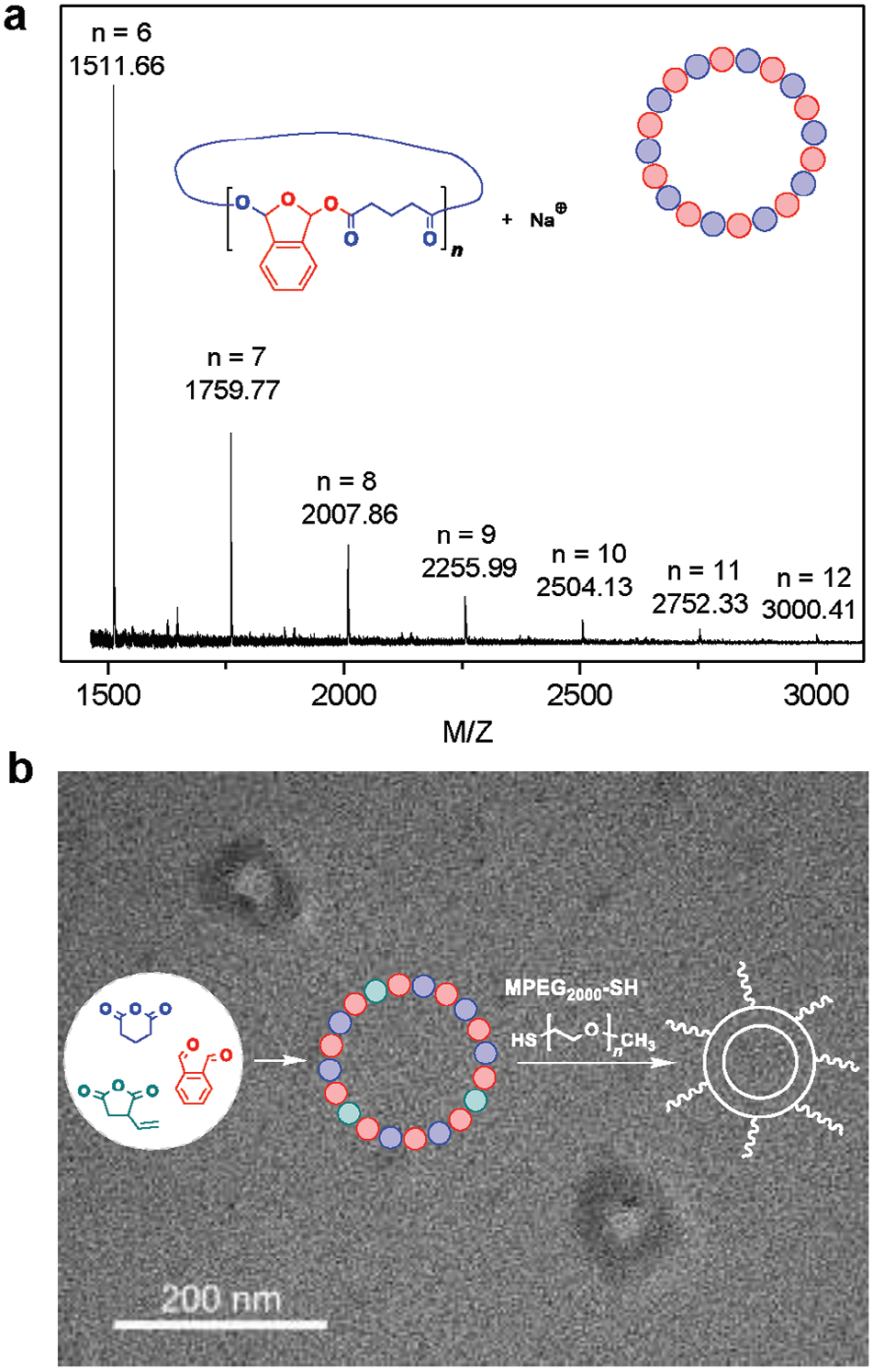
Characterization of the resulting polyester with a cyclic topology. a MOLDI‐TOF MS of the poly(OPA‐*alt*‐GA) obtained from the BF_3_·Et_2_O catalysis (entry 1 in Table 1). b) Illustration of grafting MPEG_2000_‐SH on the cyclic polyester and the TEM graph of the grafted cyclic polyester.

To further confirm the structure of the obtained poly(OPA‐*alt*‐GA), we then carried out the analysis of matrix‐assisted laser desorption/ionization time‐of‐flight mass spectroscopy (MALDI‐TOF MS). As shown in **Figure** **2**a and Figure S2 (Supporting Information), the sole distribution of [(OPA + GA)*_n_* + Na^+^] was attributed to the poly(OPA‐*alt*‐GA) with an alternating sequence as well as a cyclic topology. The *M*_n_ values of the polymers determined by GPC are higher than those illustrated by MALDI TOF because MALDI TOF discriminates against high‐*M*_n_ polymers. We also synthesized the PEG‐grafted polyester via two steps (Supporting Information): 1) the terpolymerization of OPA, GA, and 3‐vinyldihydrofuran‐2,5‐dione yielded the polyester (*M*_n_ = 3.1 kDa, *Ð* = 1.4) bearing the C═C bond on the side chain; 2) MPEG_2000_‐SH (*M*_n_ = 2.0 kDa) was then grafted on the obtained polyester via the efficient thiol‐ene click reaction. Almost all the vinyl groups in the copolymer were consumed according to the ^1^H NMR spectrum (Figure S3, Supporting Information), yielding the PEG‐grafted polymer with *M*_n_ of 4.1 kDa and *Ð* of 1.3. The diffusion‐ordered (DOSY) NMR spectrum shows a single diffusion coefficient (Figure S4, Supporting Information), suggesting the grafted structure of the polymer. The visualization of the grafted polyester was then performed by transmission electron microscopy (TEM). As shown in Figure 2b, the cyclic topology was clearly observed in the TEM image, in which the inner and outer diameters of the cyclic polymer were determined as ≈37 and ≈87 nm, respectively. Additionally, according to the ^1^H and ^13^C NMR spectra (Figure S1, Supporting Information) of the poly(OPA‐*alt*‐GA), we did not observe any peaks attributed to possible terminals. The above results indicate that the alternating copolymerization of OPA and cyclic anhydride is an efficient method for the preparation of cyclic polyesters with a high cyclic purity.

Neither OPA nor GA were homopolymerized at 25 °C with the catalysis of BF_3_·Et_2_O by the control experiments (entries 11 and 12 in Table 1). GA is unable to be homopolymerized due to thermodynamic prohibitions.^[^ ^56^ ^]^ OPA is, to date, the only known aromatic aldehyde that can be homopolymerized via chain‐growth polymerization.^[^ ^57^ ^]^ However, the OPA homopolymerization should be carried out at a lower temperature than its ceiling temperature (*T*_c_) of −43 °C,^[^ ^58^ ^]^ which has been widely investigated.^[^ ^59^, ^60^, ^61^, ^62^, ^63^ ^]^ Moore and coworkers reported that the cationic OPA homopolymerization can generate the cyclic poly(OPA) without end‐capping.^[^ ^64^ ^]^ Based on the cationic macrocyclization mechanism demonstrated by Moore et al.,^[^ ^60^ ^]^ we proposed that the cyclic poly(OPA‐*alt*‐GA) was mainly generated from the back‐biting reaction (Figure S5, Supporting Information). The back‐biting can occur at any position along the polymer chain to produce a cyclic copolymer, but the ring closure from only the terminal position is shown, resulting in the lower measured *M*_n_ by GPC than the theoretical *M*_n_ of the polymer. The purified cyclic polymer could be further ring‐expanded by adding another monomer. The *M*_n_ of the purified poly(OPA‐*alt*‐GA) was successfully extended from 12.8 to 24.9 kDa by the addition of another portion of monomer (Figure S6, Supporting Information), which is similar to the results reported by Moore and coworkers.^[^ ^60^, ^65^ ^]^ The non‐ideal unimodal curve of the chain‐extension polymer may be due to the non‐participation of some precursor polymer. Consequently, although neither OPA nor GA can be homopolymerized at room temperature, their copolymerization with the cationic mechanism exhibits an alternating manner.

**Figure 3:**
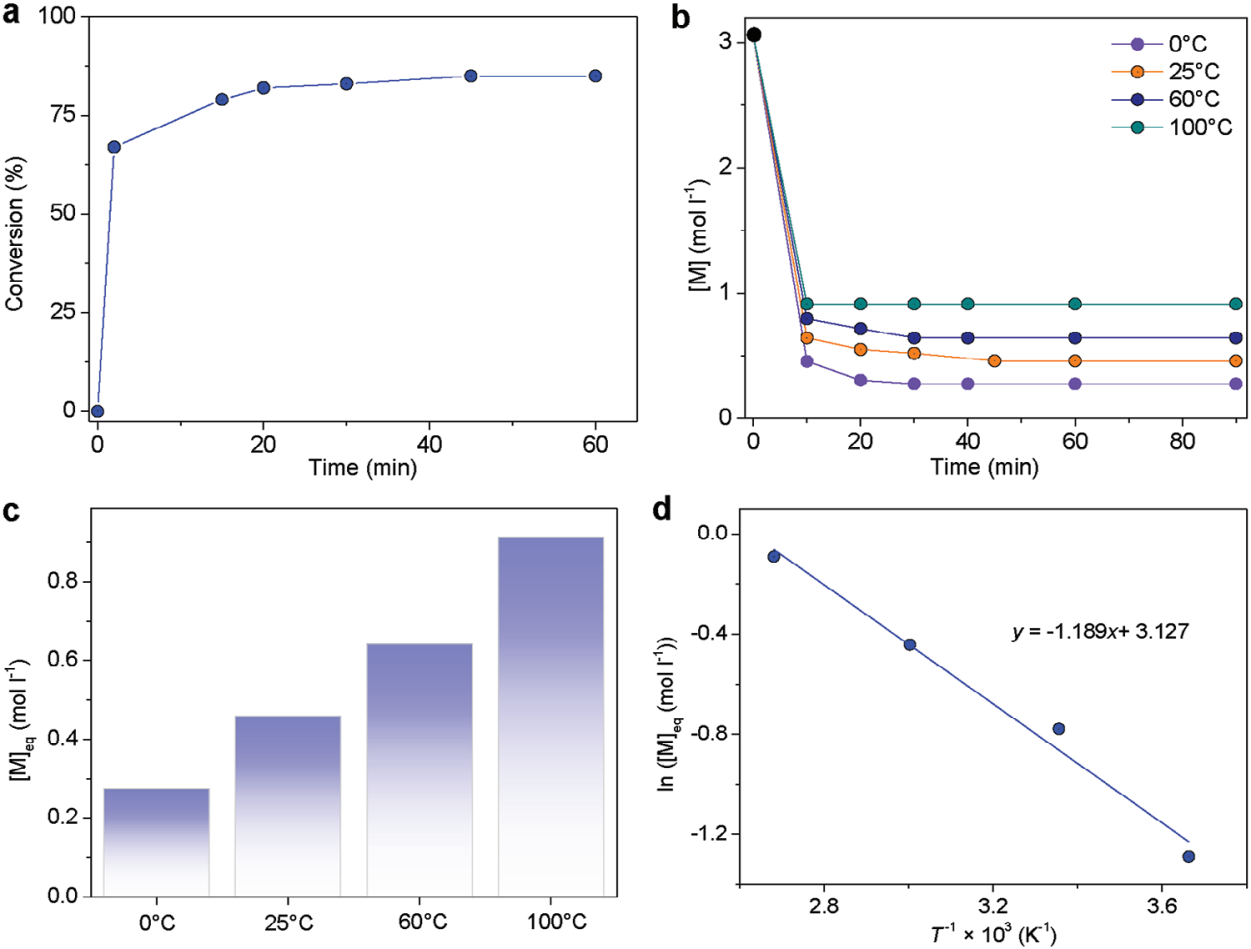
Kinetics study of the OPA and GA copolymerization, [OPA]_0_ = 3.06 m in CH_2_Cl_2_, [OPA]_0_:[GA]_0_:[BF_3_·Et_2_O] = 100:100:1. a) Plots of monomer conversion versus time as monitored by ^1^H NMR spectroscopy, at 25 °C. b) Plots of monomer concentration versus time as monitored by ^1^H NMR spectroscopy. c Equilibrium monomer concentration at different temperature conditions. d) Van't Hoff plot of ln[M]_eq_ versus the reciprocal of the absolute temperature (*T* ^−1^).

Interestingly, by kinetic and thermodynamic studies, the copolymerization of OPA and GA was manifested to be highly reversible, which would be unavailable in previously reported copolymerization of epoxide and cyclic anhydride.^[^ ^41^ ^]^ With BF_3_·Et_2_O as the catalyst, at 25 °C, the monomer conversion was up to 67% within 2 mins and remained almost constant at 82 – 85% from 20 to 60 mins (**Figure** **3**a). The equilibrium monomer concentrations ([OPA]_eq_ = [GA]_eq_) were tested as a function of temperatures (Figure 3b,c), which are 0.28, 0.46, 0.64, and 0.91 m at 0, 25, 60, and 100 °C, respectively. The Van't Hoff plot of ln[M]_eq_ versus 1/*T* showed a straight line with a slope of −1.189 and an intercept of 3.127 (Figure 3d). Based on the equation of ln[M]_eq_ = Δ*H*°/*RT* – Δ*S*°/*R*, the parameters were calculated to be Δ*H*°_OPA+GA_ = −9.9 kJ mol^−1^ and Δ*S*°_OPA+GA_ = −26.0 J mol^−1^ K^−1^. Subsequently, based on the equation of *T*_c_ = Δ*H*°/(Δ*S*°+ *R* ln[M]_0_), the *T*_c_°_OPA+GA_ was calculated to be 108 °C at [OPA]_0_ = [GA]_0_ = 1 m (in CH_2_Cl_2_). For comparison, by previous studies,^[^ ^58^ ^]^ the thermodynamic parameters of the OPA homopolymerization were calculated to be Δ*H*°_OPA_ = −22.2 kJ mol^−1^, Δ*S*°_OPA_ = −96.3 J mol^−1^ K^−1^, and *T*_c_°_OPA_ = −43 °C. Thus, the OPA and GA alternating copolymerization is thermodynamically more favorable than the OPA homopolymerization.

**Figure 4:**
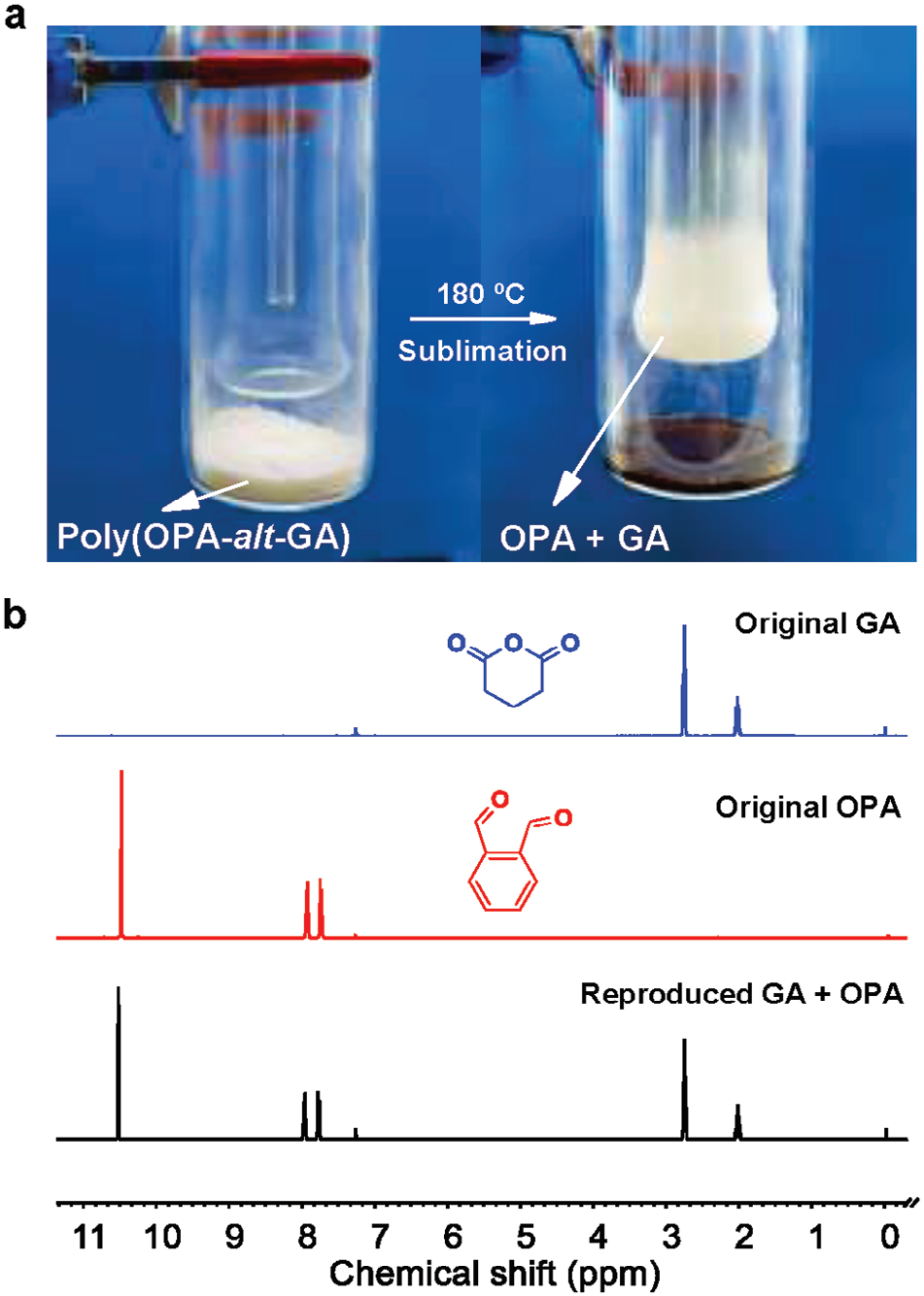
Illustration of the chemical recovery of poly(OPA‐*alt*‐GA). a) Depolymerization of poly(OPA‐*alt*‐GA) to monomers by the sublimation operation. b ^1^H NMR spectra in CDCl_3_ of the initial GA (blue line) and OPA (red line) monomers and the regenerated monomers (black line).

Based on the chemical reversible manners of the copolymerization, we next carried out the chemical recovery of the poly(OPA‐*alt*‐GA). With the simple sublimation operation (**Figure** **4**a), at 180 °C, for 10 h, under vacuum, without any solvents or catalysts, under dark conditions, 3.0 g of the poly(OPA‐*alt*‐GA) (*M*_n_ = 13.2 kDa, *Đ* = 1.4) was converted into 2.8 g of the mixture containing [OPA]:[GA]:[glutaric acid]:[isobenzofuran‐1(3H)‐one] = 1.00:1.14:0.16:0.12 (Figure S7, Supporting Information). Glutaric acid was generated by the hydrolysis of GA. Isobenzofuran‐1(3H)‐one was produced from the oxidation of OPA and was greatly suppressed under dark conditions. The second sublimation operation of the mixture at 60 °C yielded 2.1 g (70% yield) of the mixture containing only OPA and GA ([OPA]:[GA] = 1:0.79), as determined by ^1^H NMR spectrum (Figure 4b). Then, in the absence of further purification, at 25 °C for 1 h, the addition of BF_3_·Et_2_O ([GA]:[BF_3_·Et_2_O] = 100:1) to the mixture successfully initiated the copolymerization, yielding 1.5 g of poly(OPA‐*alt*‐GA) with *M*_n_ of 13.0 kDa and *Đ* of 1.4 (Figure S8, Supporting Information). Non‐equal amounts of GA and OPA cause a slight difference in the molecular weight of the copolymer from that before depolymerization. Overall, the closed‐loop chemical recycling of the polymer can be easily carried out at high temperatures by simple sublimation operations. Additionally, the polymer can be completely depolymerized into monomers at a lower temperature in solution. With a concentration of 0.01 m in CH_2_Cl_2_, at 100 °C for 2 h, and using 1 mol% BF_3_·Et_2_O as a depolymerization catalyst, the poly(OPA‐*alt*‐GA) was completely depolymerized into the monomers of OPA and GA (Figure S9, Supporting Information).

We also extended the method to the bromine‐substituted OPA (Br‐OPA) and other 4 commercially available cyclic anhydrides (entries 13–17, Table 1), including 3‐methylglutaric anhydride (MGA), 3,3‐dimethylglutaric anhydride (DMGA), 3‐isobutyl‐glutaric anhydride (*i*BuGA), and thiodiacetic anhydride (TDGA). At 25 °C, for 1 h, with the feeding ratio of [M1]:[M2]:[BF_3_·Et_2_O] = 100:100:1, the copolymerization yields a series of polyesters with *M*_n_ of 4.4 – 6.3 kDa (Figure S10, Supporting Information), *Ð* of 1.3 – 1.5, and well‐defined alternating sequences determined by NMR analysis (Figures S11–S15, Supporting Information). Taking the poly(OPA‐*alt*‐TDGA) as an example, the high‐purity cyclic topology was revealed by the MALDI‐TOF MS (Figure S16, Supporting Information).

The copolymer of poly(OPA‐*alt*‐GA) exhibits a glass transition temperature (*T*_g_) of 67 °C, as determined by differential scanning calorimetry (DSC, Figure S17, Supporting Information). When an alkyl side group is introduced into the polymer chain, the *T*_g_ of the copolymer decreases, such as poly(OPA‐*alt*‐MGA) with *T*_g_ of 58 °C (Figure S18, Supporting Information), poly(OPA‐*alt*‐DMGA) with *T*_g_ of 62 °C (Figure S19, Supporting Information), and poly(OPA‐*alt*‐*i*BuGA) with *T*_g_ of 40 °C (Figure S20, Supporting Information). Both the sulfur‐ and bromine‐containing copolymers of poly(OPA‐*alt*‐TDGA) and poly(Br‐OPA‐*alt*‐GA) exhibit *T*_g_ of 52 °C (Figures S21,S22, Supporting Information). The polyesters exhibit thermal decomposition temperatures (*T*_d_, temperature at 5% decomposition) of 177 – 203 °C, as determined by thermogravimetric analysis (TGA, Figures S17–S22). Through the stress‐strain experiment, the specimens of the poly(OPA‐*alt*‐GA) (entry 9 in Table 1) display the ultimate tensile strength (σ_B_) of 7.3 ± 1.1 MPa and the elongation at break (ɛ_Β_) of 2.3 ± 0.2% (Figure S23, Supporting Information). Additionally, the polymers are acid‐sensitive owing to the incorporation of an in‐chain acetal group. As an example, after being placed in an aqueous HCl solution (2 m) for 24 h, the *M*_n_ of the poly(OPA‐*alt*‐GA) was reduced from 11.8 kDa to 5.9 kDa (Figure S24, Supporting Information). The structure and properties of these polymers have a lot of room for adjustment using two distinct monomer sets.

## 3 Conclusion

In summary, we have demonstrated a new and facile method for the synthesis of cyclic and recyclable polyesters using commercially available monomers of OPA and cyclic anhydrides. The copolymerization of cyclic anhydride and OPA with the cationic mechanism exhibits alternating and reversible characteristics. The method is performed under mild conditions and produces polyesters in high yields within 1 h. The resulting polyesters possess well‐defined alternating sequences, high‐fidelity cyclic topology, an easy‐to‐tune structure using two distinct monomer sets, and practical chemical recyclability. The current polyesters are acid‐degraded and have relatively poor thermal stability. Ongoing works will focus on the development of controllable catalysts, further clarification of the mechanism, and finding applications in some specific fields such as sacrificial materials.

## Conflict of Interest

The authors declare no conflict of interest.

## Supporting information

Supporting Information

## Data Availability

The data that support the findings of this study are available in the supplementary material of this article.
