# Supplementary material for: Cyclic Polyesters with Closed‐Loop Recyclability from A New Chemically Reversible Alternating Copolymerization
Source: Adv Sci (Weinh). 2023 Nov 30;11(4):2306072. doi: 10.1002/advs.202306072 (PMC10811513; doi:10.1002/advs.202306072)
Supplement: Supplementary file 1 — Supporting Information [file ADVS-11-2306072-s001.pdf]

ADVANCED SCIENCE
Open Access

# Supporting Information

for *Adv. Sci.*, DOI 10.1002/advs.202306072

Cyclic Polyesters with Closed-Loop Recyclability from A New Chemically Reversible Alternating Copolymerization

*Xiaoxian Lu, Xun Zhang, Chengjian Zhang* and Xinghong Zhang**

# *Supporting Information*

# Cyclic polyesters with closed-loop recyclability from a new chemically reversible alternating copolymerization

Xiaoxian Lu, Xun Zhang, Chengjian Zhang, and Xinghong Zhang

National Key Laboratory of Biobased Transportation Fuel Technology, International Research Center for X Polymers, Department of Polymer Science and Engineering, Zhejiang University, Hangzhou 310027 (China)

**Corresponding authors:** chengjian.zhang@zju.edu.cn (Chengjian Zhang); xhzhang@zju.edu.cn (Xinghong Zhang)

# Table of Contents

**Materials and methods** ....................................................................................... S3

**Figure S1.** (a) $^1$H NMR and (b) $^{13}$C NMR spectra of poly(OPA-*alt*-GA)............................. S5

**Figure S2.** MALDI-TOF MS of cyclic poly(OPA-*alt*-GA) .................................................. S6

**Figure S3.** $^1$H NMR spectra of the copolymers before and after grafted............................... S7

**Figure S4.** DOSY NMR spectrum of the PEG-grafted copolymer ........................................ S8

**Figure S5.** Mechanism of cyclic polymer formation by back-biting reaction ........................ S9

**Figure S6.** GPC curves of the poly(OPA-*alt*-GA) before and after ring expansion............. S10

**Figure S7.** $^1$H NMR spectra of the product from the first sublimation ................................ S11

**Figure S8.** GPC curves of the poly(OPA-*alt*-GA) before and after chemical recycle. ......... S12

**Figure S9.** $^1$H NMR spectrum of the degradation product in low concentration................. S13

**Figures S10.** GPC curves of the obtained polymers ........................................................... S14

**Figures S11-S15.** (a) $^1$H NMR and (b) $^{13}$C NMR spectra of the obtained polymers ............ S15

**Figure S16.** MALDI-TOF MS of cyclic poly(OPA-*alt*-TDGA) .......................................... S20

**Table S1.** $T_g$ and $T_d$ values of the obtained polymers .......................................................... S21

**Figures S17-S22.** DSC and TGA curves of the obtained polymers ..................................... S22

**Figure S23.** Stress-strain curves of the poly(OPA-*alt*-GA) (entry 9 in Table 1).................. S28

**Figure S24.** GPC curves of the poly(OPA-*alt*-GA) before and after stirred in 2 M HCl solution for 24 h. ............................................................................................................... S29

## Materials and methods

### Materials

*o*-Phthalaldehyde, 4-bromophthalaldehyde, and $MPEG_{2000}$-SH ($M_n$ = 2 kDa) were purchased from bidepharmatech (Shanghai). Glutaric anhydride, 3-methylglutaric anhydride, 3,3-dimethylglutaric anhydride, 3-isobutyl-glutaric anhydride and thiodiacetic anhydride were purchased from Aladdin Reagent Company (Shanghai) and sublimated before use. $BF_3$•$Et_2O$, $InCl_3$, $InBr_3$, $NH(OTf)_2$, TfOH, and $H_2SO_4$ were purchased from Sigma Aldrich Chemical Co. and used as received.

### Characterization methods

$^1$H and $^{13}$C NMR spectra were recorded on a Bruker Advance DMX 400 MHz spectrometer. Chemical shift values were referenced to $CHCl_3$ as internal standard at 7.26 ppm or TMS as internal standard at 0 ppm for $^1$H NMR (400 MHz) and against $CDCl_3$ at 77.16 ppm for $^{13}$C NMR (100 MHz).

The molecular weights and molecular weight distributions of polymers were determined with a PL-GPC220 chromatograph (Polymer Laboratories Ltd) equipped with an HP 1100 pump from Agilent Technologies. The GPC columns were eluted with THF at 1.0 ml/min at 35 °C. The sample concentration was 0.4 wt. % and the injection volume was 50 μL. Calibration was performed using monodisperse polystyrene standards covering the molecular weight range from 580 to 460 000 Da. The absolute molecular weight of the polymers were obtained by a PL-GPC 220 chromatograph coupled with triple detectors (refractive index, intrinsic viscosity, and light scattering detectors (laser wavelength, λ = 660 nm)) eluted using THF with 1mL/min at 35 °C.

Matrix-assisted laser desorption/ionization time-of-flight (MALDI-TOF) mass spectrometric measurements were performed on a Bruker Ultraflex MALDI TOF mass spectrometer, equipped with a nitrogen laser delivering 3 ns laser pulses at 337 nm. Trans-2-[3-(4-tert-butylphenyl)-2-methyl-2-propenylidene]malononitrile was used as the matrix. Sodium trifluoroacetate was added for ion formation.

The decomposition temperature ($T_d$) of the polymers were determined by using TA Q50 instrument. The sample was heated from 40 to 400 °C at a rate of 10 °C/min under nitrogen atmosphere. Temperature when the mass loss is five percent was taken as $T_{d,5\%}$.

Differential scanning calorimetry (DSC) was taken on a DSCQ200 equipped with a liquid nitrogen cooling system. 3~5 mg of samples were placed in aluminum pans. The cooling and heating rates were 10 °C/min. The viscosity measurement was carried out using a rotational viscometer (DV2T Touch Screen Viscometer) purchased from Brookfield.

The cyclic structures were imaged by transmission electron microscopy (TEM) conducted on Hitachi 7700 at an acceleration voltage of 120 kV. To prepare TEM samples, 3 μL of the polymer solution (1 mg/mL in $CH_2Cl_2$) was dropped onto a carbon-coated copper grid and dried.

**Representative procedure for copolymerization**

All syntheses were carried out in a nitrogen-filled glovebox. A 10 ml reaction tube with a magnetic stirrer was dried in an oven at 120 °C overnight and transferred into the glovebox immediately. The copolymerization below is taken from entry 1 in Table 1 as an example. Glutaric anhydride (1.53 mmol), *o*-phthalaldehyde (1.53 mmol), $CH_2Cl_2$ (0.5 ml) and $BF_3$•$Et_2O$ (0.0153 mmol) were added into the reaction tube successively. The reaction tube was sealed with a Teflon-lined cap and was stirred at 25 °C for 1 h. Sodium phenolate was added to terminate the reaction. After polymerization, the crude product was dissolved in dichloromethane and precipitated from methanol three times. The obtained precipitate was collected and dried under vacuum at 45 °C.

**Synthesis of PEG-grafted copolymer**

Glutaric anhydride (1.53 mmol), allylsuccinic anhydride (1.53 mmol), *o*-phthalaldehyde (3.06 mmol), $CH_2Cl_2$ (0.5 ml) and $BF_3$•$Et_2O$ (0.0306 mmol) were added into the reaction tube successively. After the reaction had been carried out at room temperature for 3 h, the mixture was precipitated into methanol three times. 0.2 g of the resulting copolymer ($M_n$ = 3000, 0.006 mmol vinyl group, 1 eq.), 0.26 g $MPEG_{2000}$-SH (0.06 mmol SH group, 10 eq.), and 2 mg of AIBN were dissolved in 3 mL $CH_2Cl_2$ and heated to 60 °C for 12 h. The obtained polymer was precipitated by methanol and dried under vacuum at 45 °C.

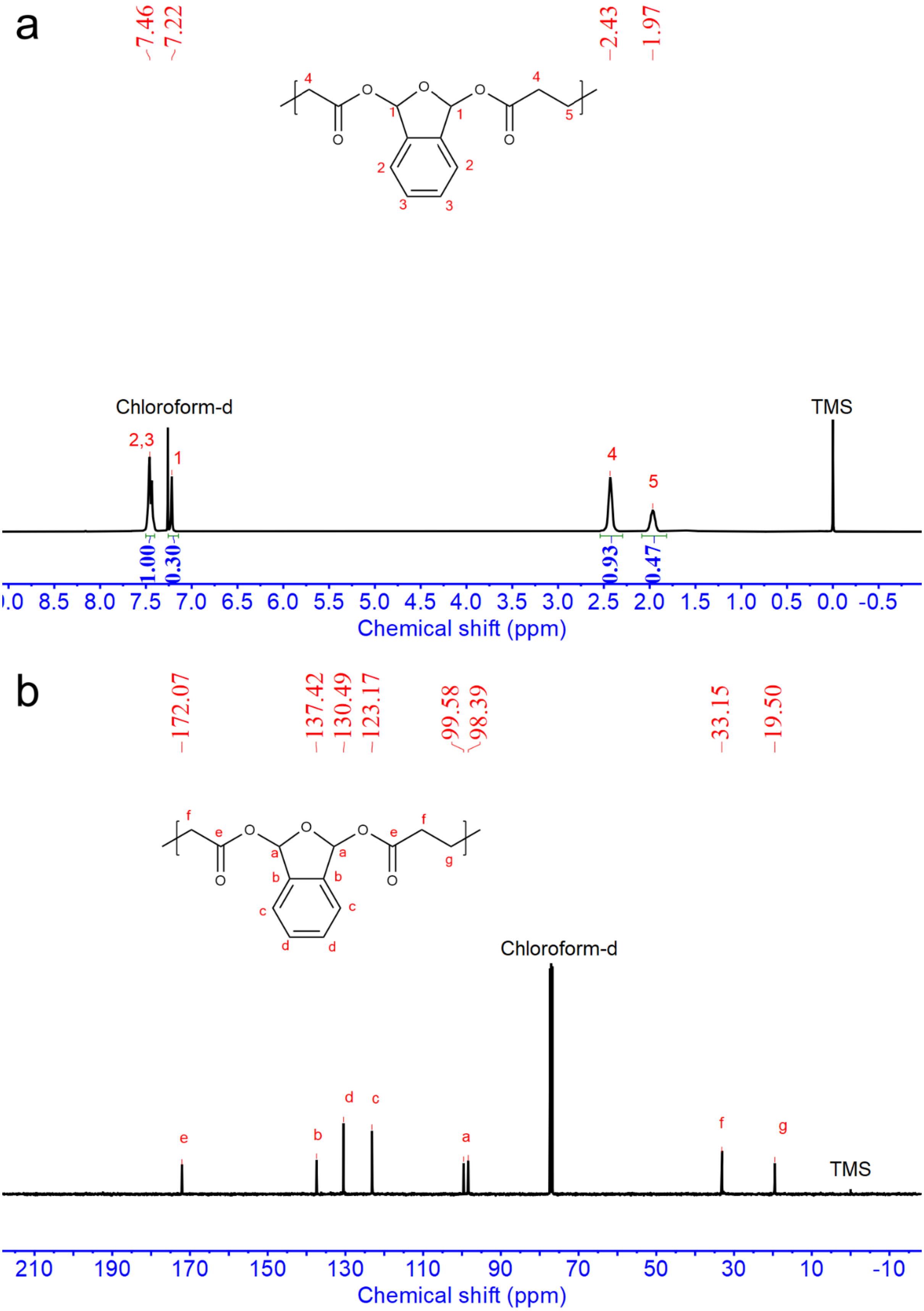


**Figure S1.** (a) $^{1}$H NMR and (b) $^{13}$C NMR spectra of poly(OPA-*alt*-GA).

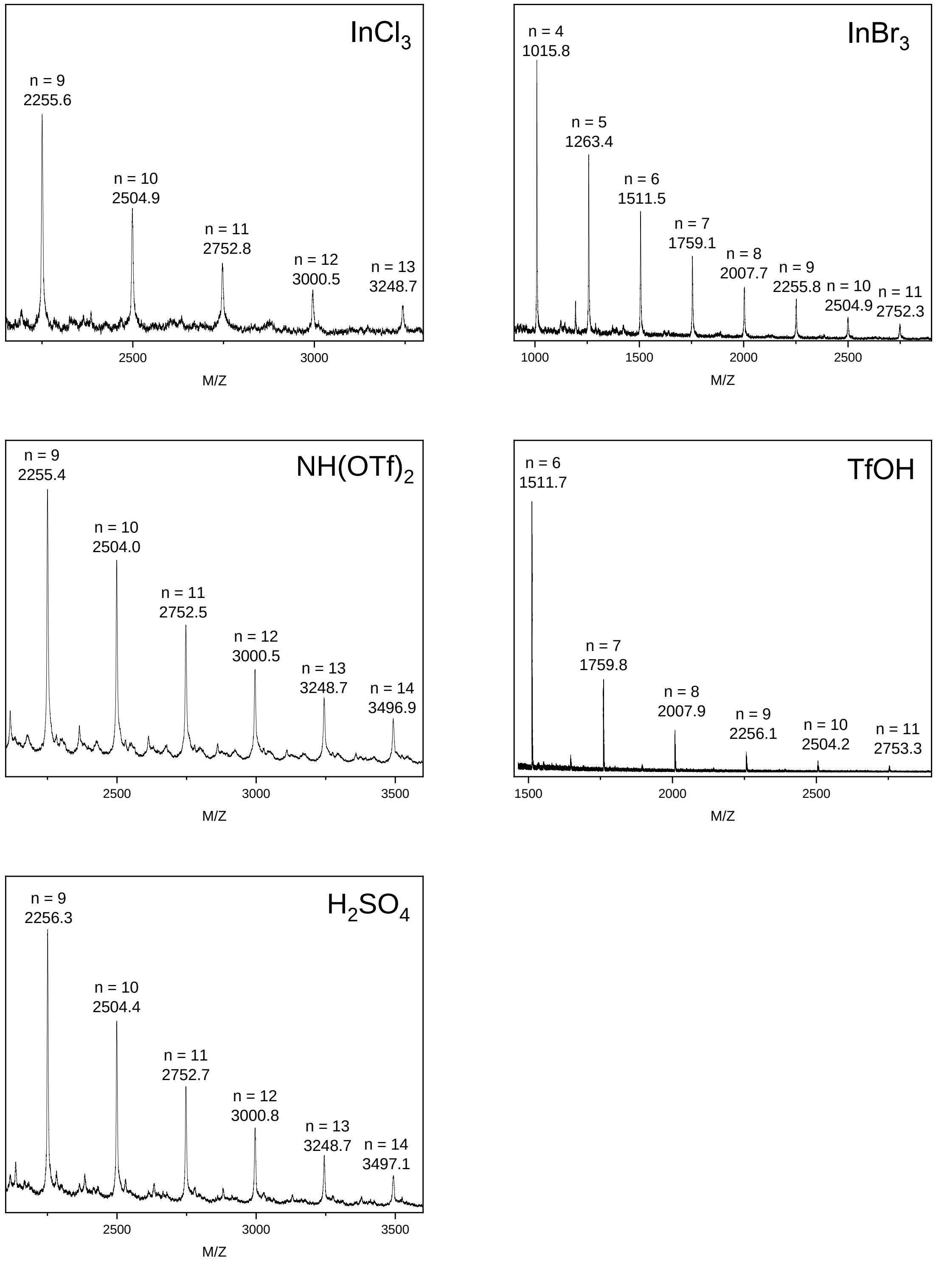


**Figure S2.** MALDI-TOF MS of cyclic poly(OPA-*alt*-GA) synthesized by different catalysts.

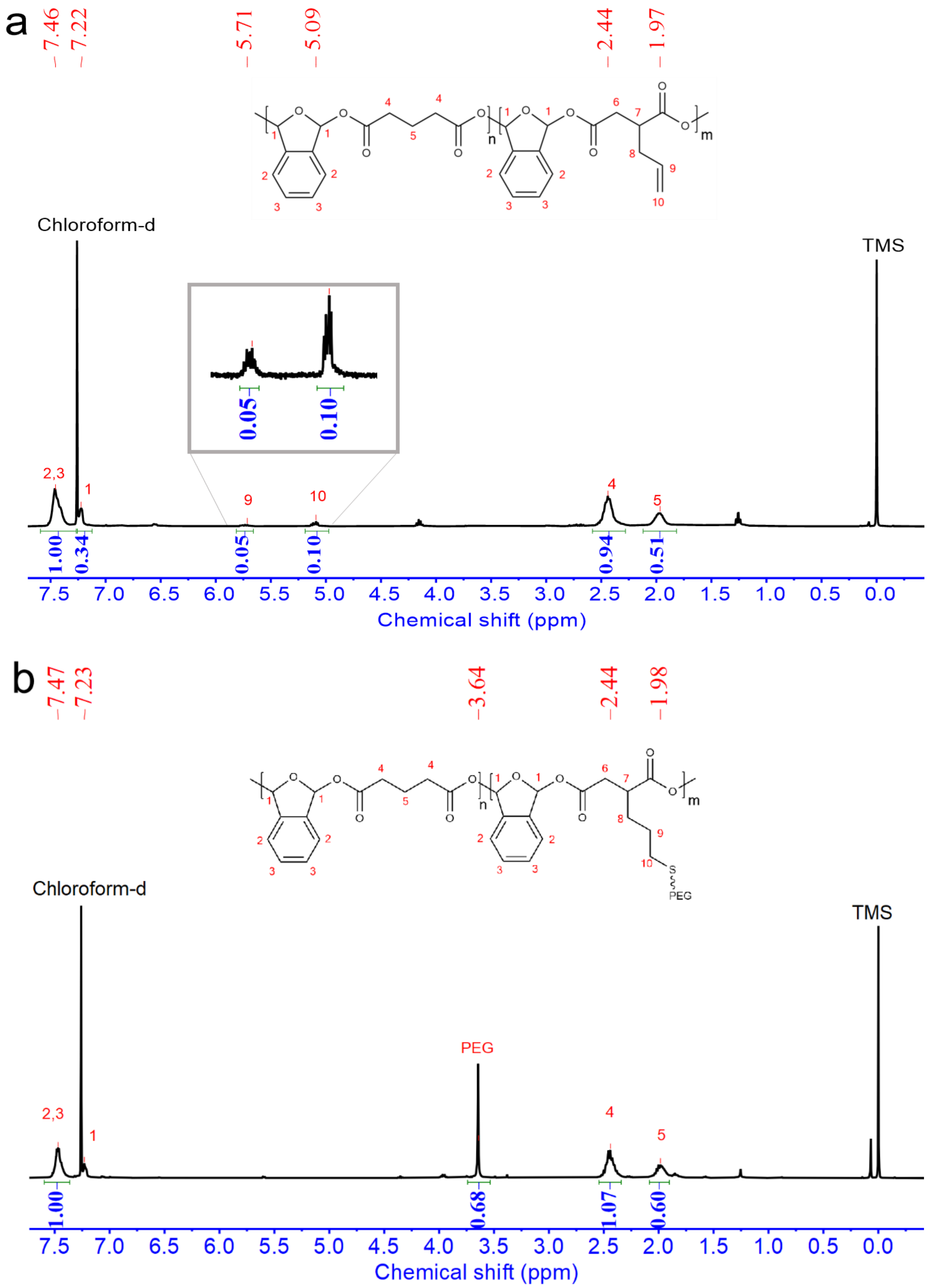


**Figure S3.** $^{1}$H NMR spectra of poly(OPA-*alt*-GA)-*random*-poly(OPA-*alt*-3-vinyldihydrofuran-2,5-dione): (a) before grafted; (b) after grafted.

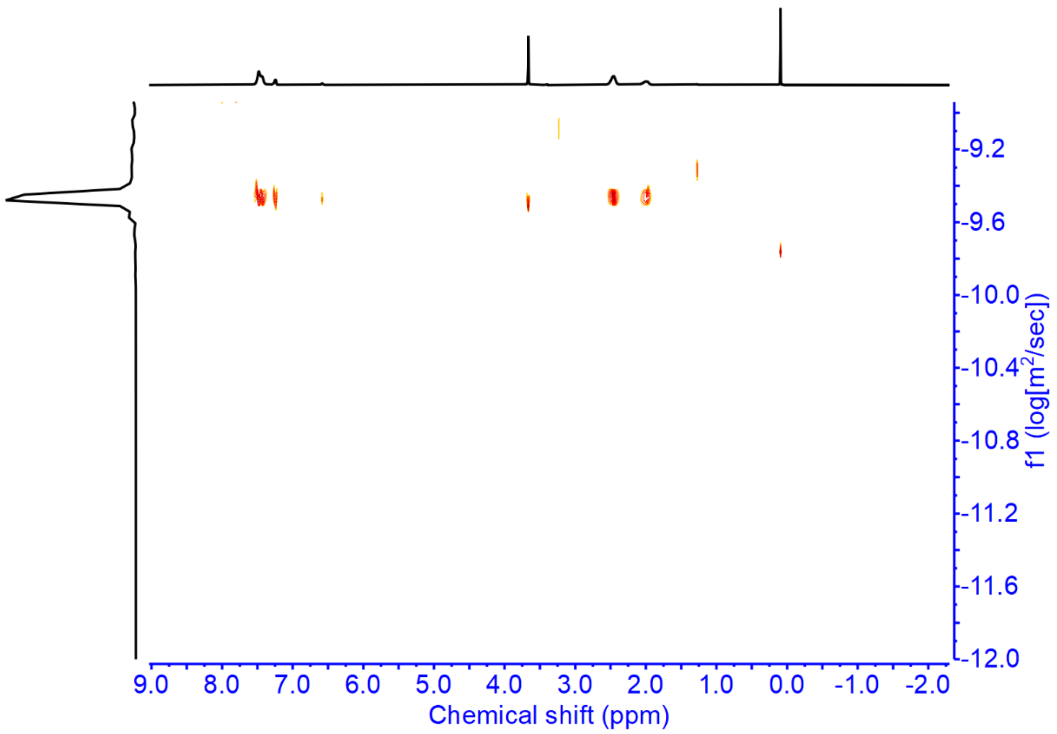


**Figure S4.** DOSY NMR spectrum of the PEG-grafted copolymer.

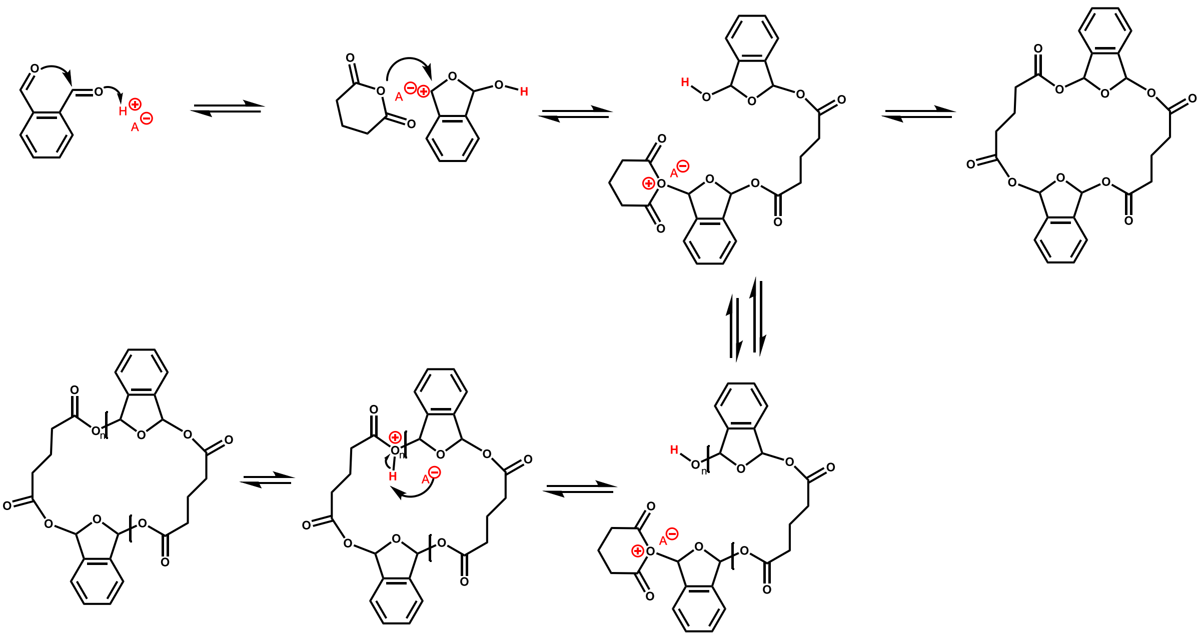

**Figure S5.** Proposed mechanism of cyclic polymer formation by back-biting reaction.

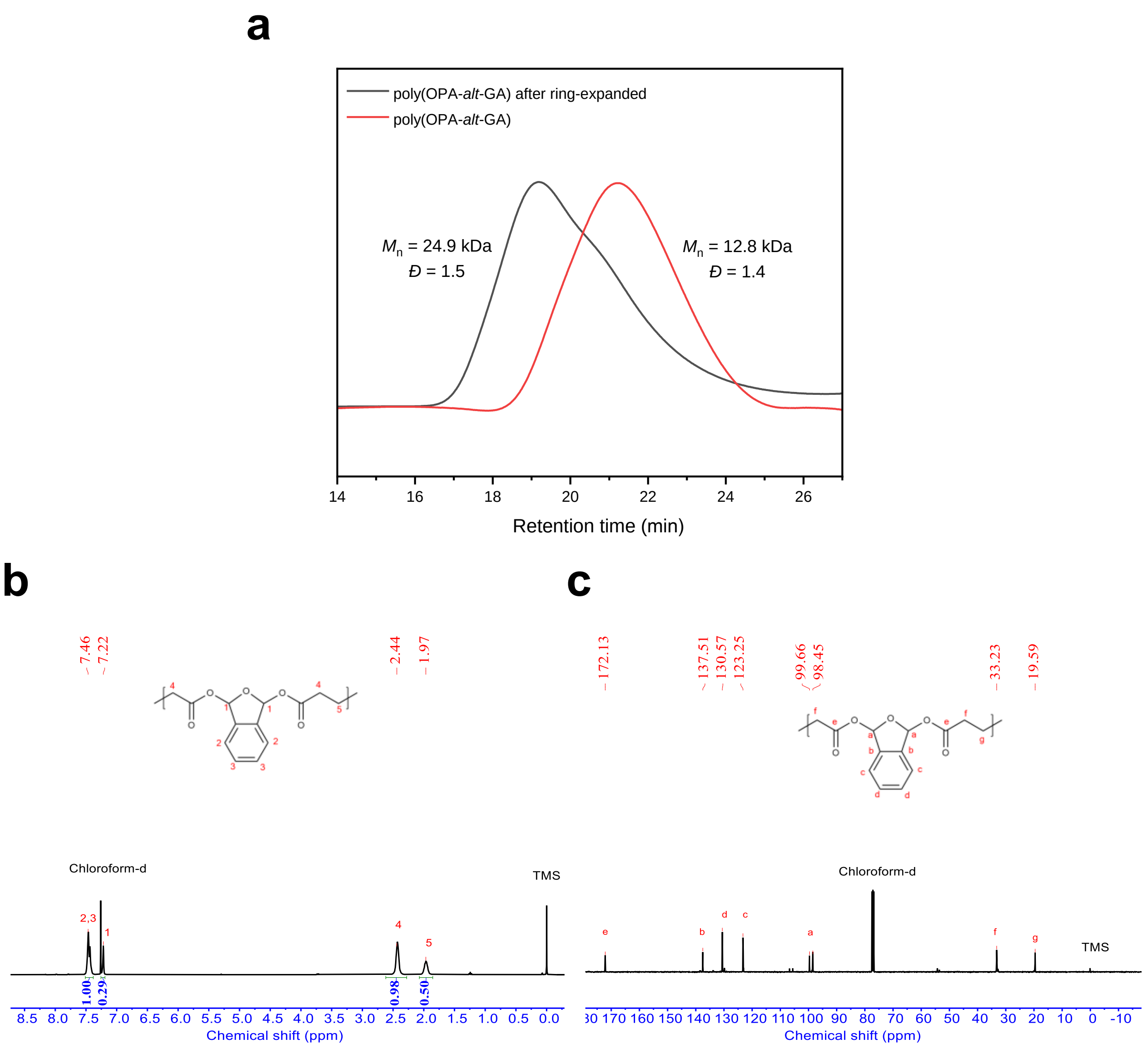


**Figure S6.** (a) GPC curves of the poly(OPA-*alt*-GA) before and after ring expansion. (b) $^{1}H$ and (c) $^{13}C$ NMR spectra of the chain-extended polymer. Reaction conditions: 0.07 g of purified poly(OPA-*alt*-GA) ($M_n$ = 12.8 kDa), 1.53 mmol GA, 1.53 mmol OPA, 0.003 mmol $InBr_3$, in 0.2 ml DCM, at room temperature for 0.5 h.

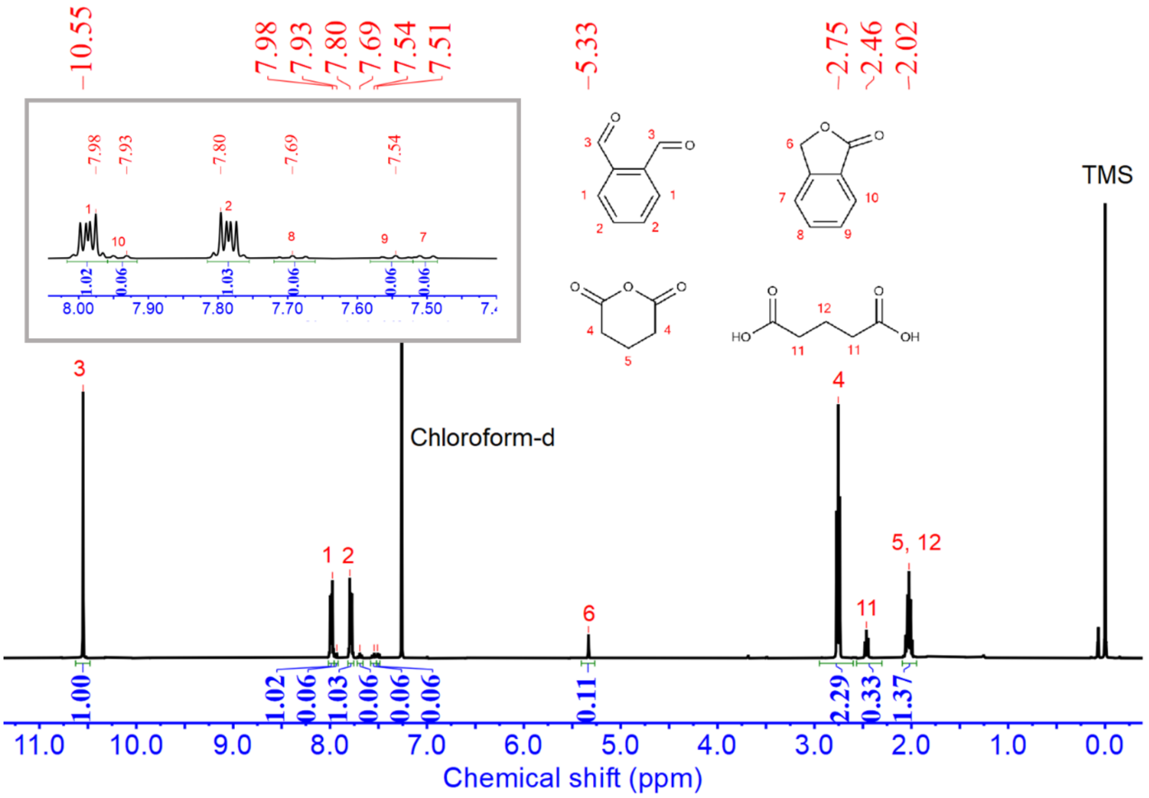


**Figure S7.** $^{1}$H NMR spectrum of the product from the first sublimation.

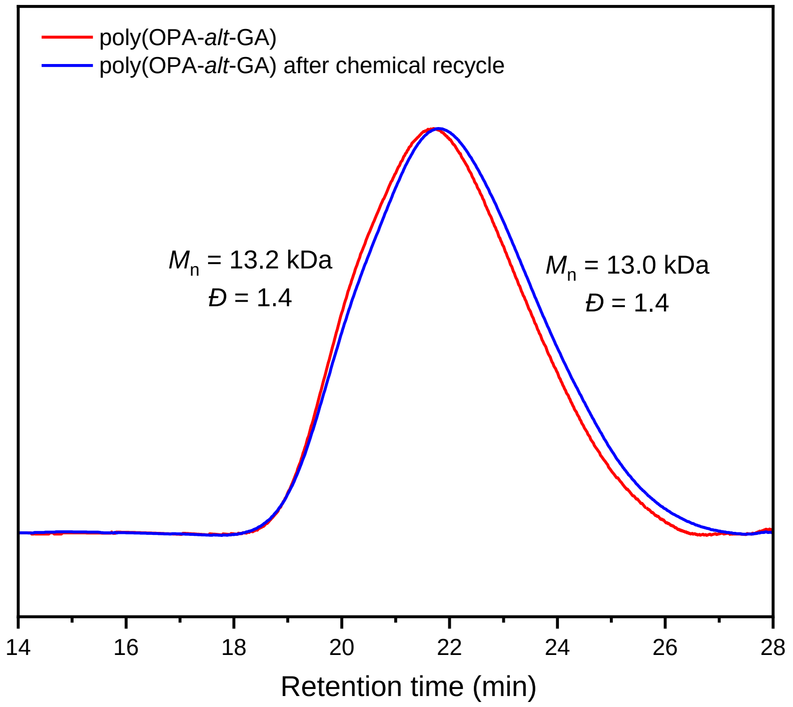


**Figure S8.** GPC curves of the original and recovered poly(OPA-*alt*-GA).

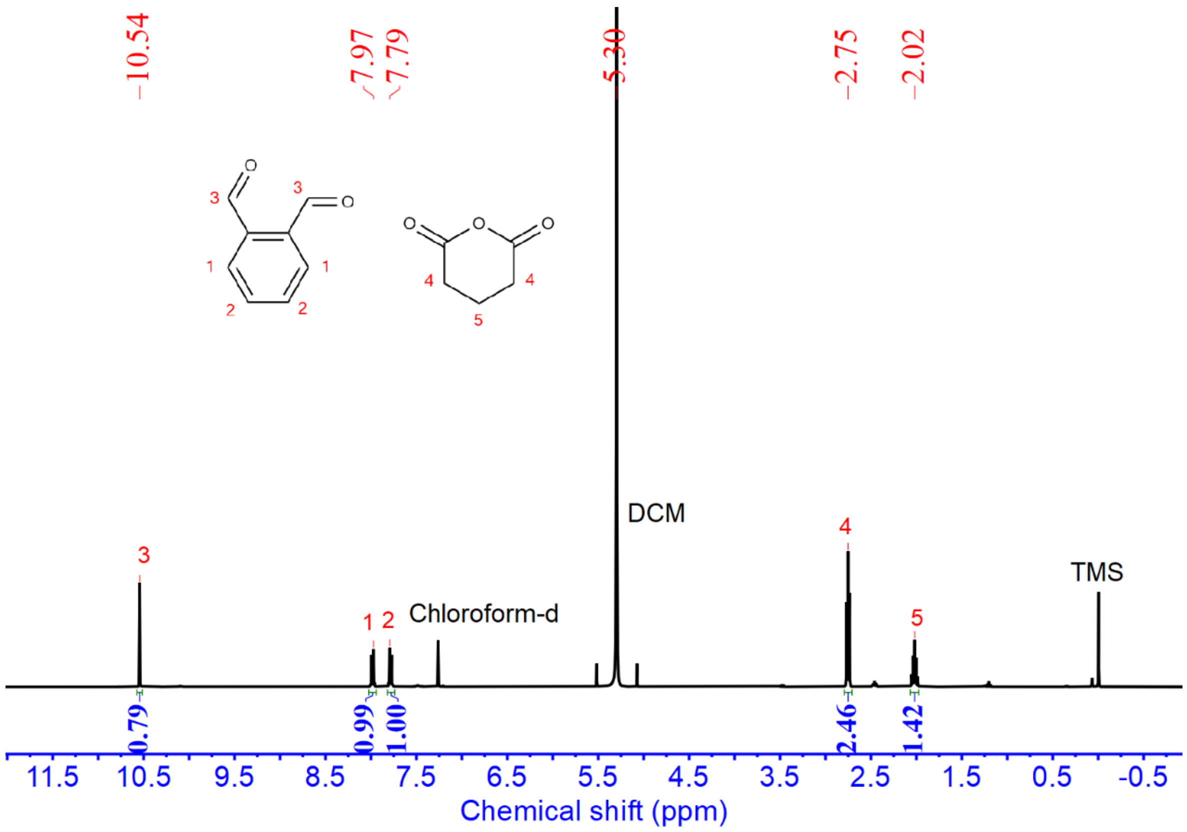


**Figure S9.** $^1$H NMR spectrum of the degradation product in low concentration (0.01 M of poly(OPA-*alt*-GA) in DCM, at 100 °C for 2 h, with 1 mol% $BF_3 \cdot Et_2O$).

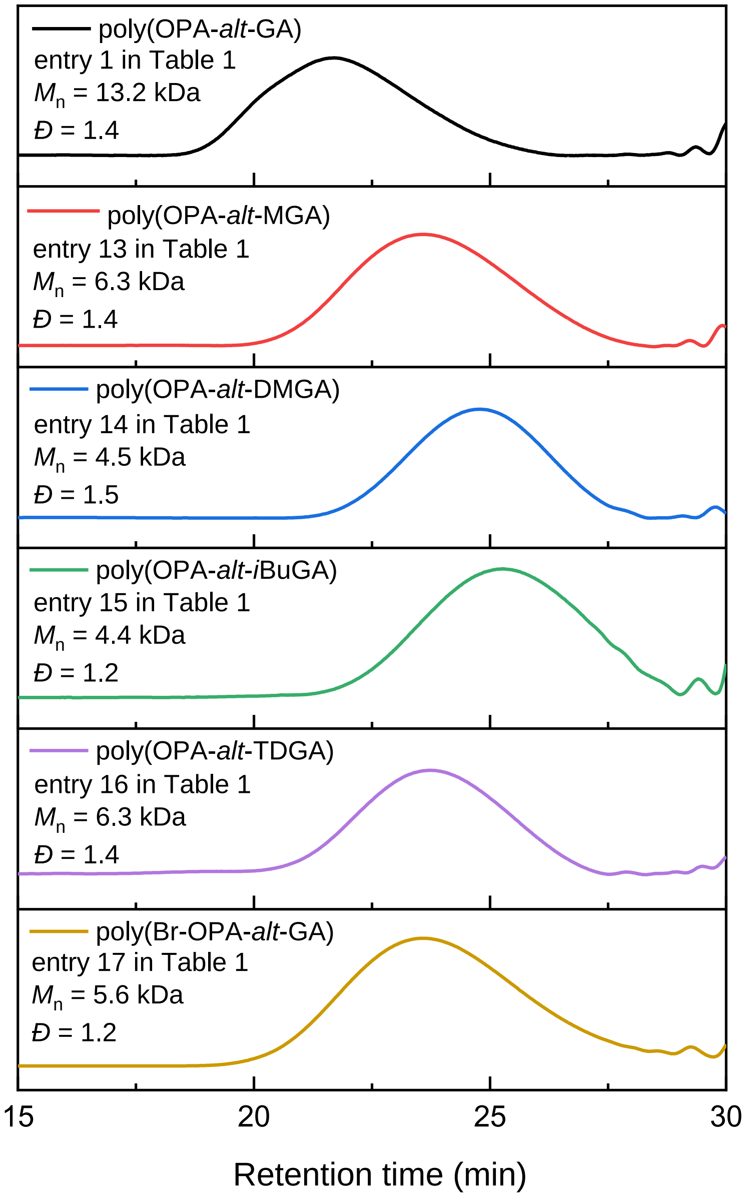


**Figure S10.** GPC curves of the obtained polymers.

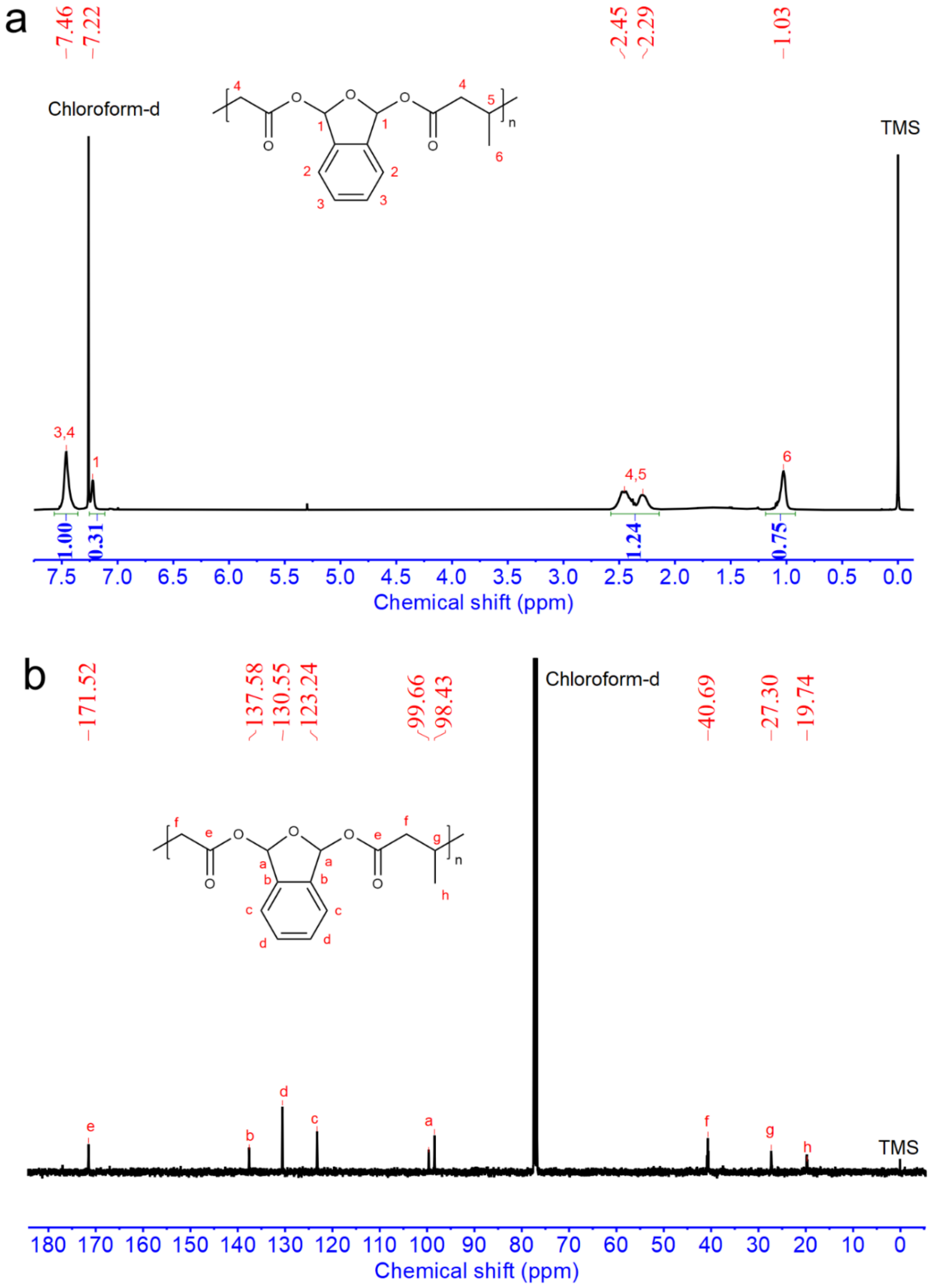


**Figure S11.** (a) $^{1}$H NMR and (b) $^{13}$C NMR spectra of poly(OPA-*alt*-MGA).

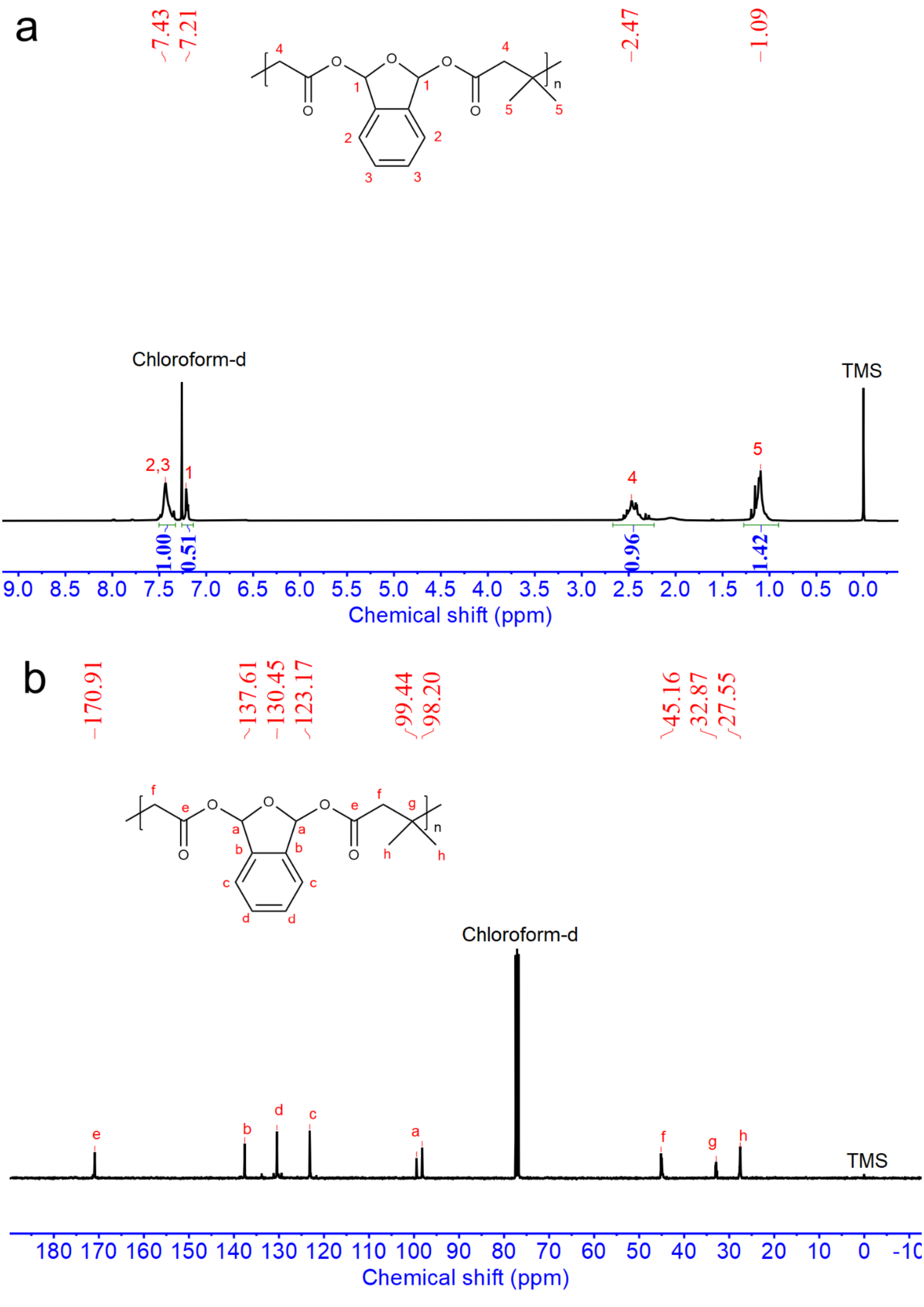


**Figure S12.** (a) $^{1}$H NMR and (b) $^{13}$C NMR spectra of poly(OPA-*alt*-DMGA).

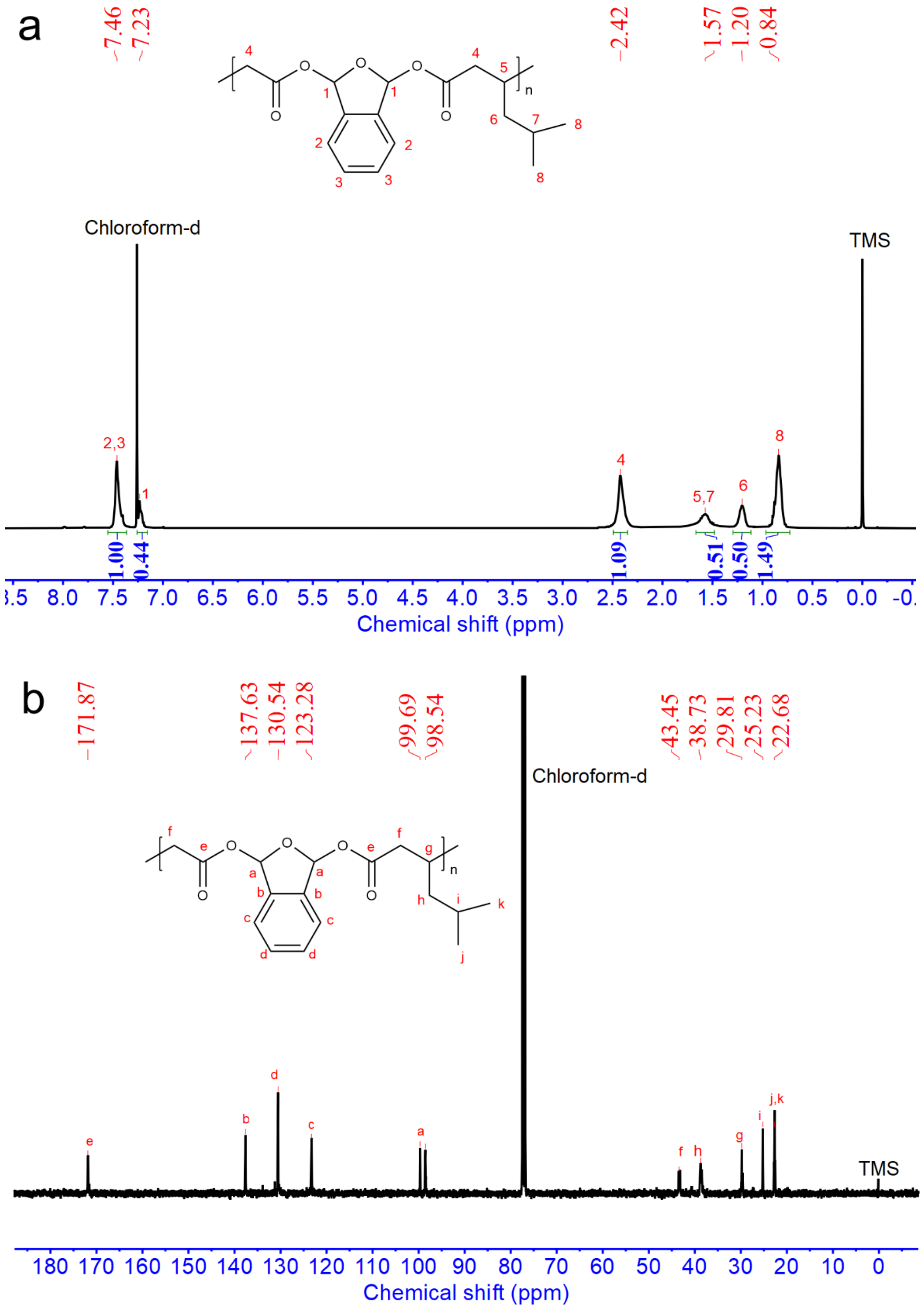


**Figure S13.** (a) $^{1}$H NMR and (b) $^{13}$C NMR spectra of poly(OPA-*alt*-*i*BuGA).

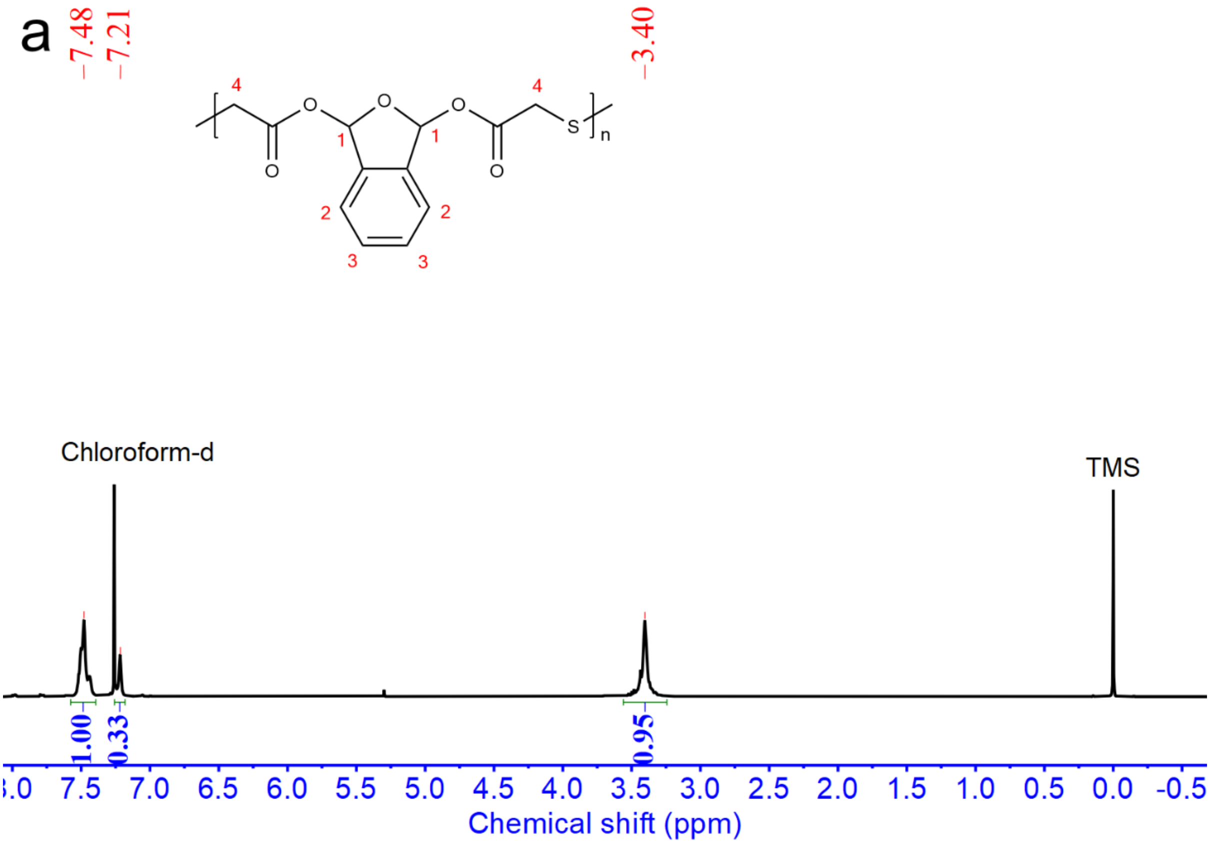


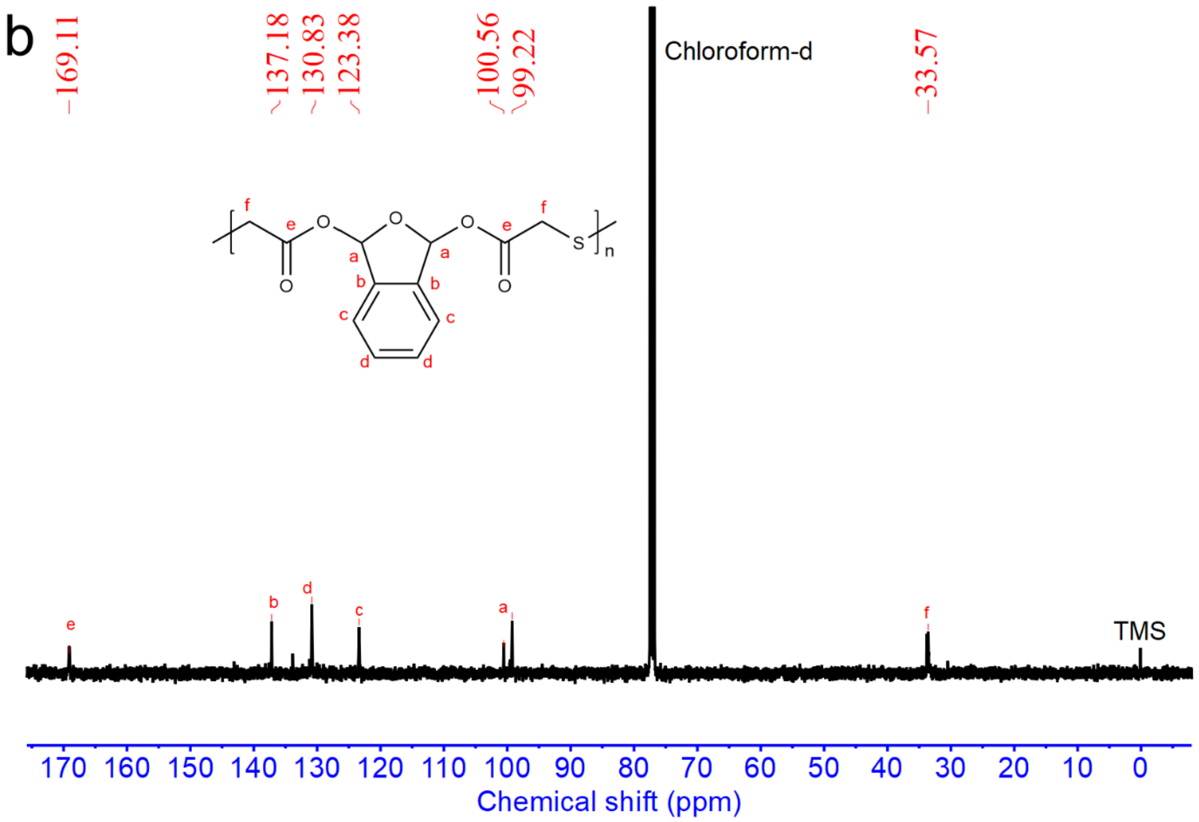


**Figure S14.** (a) $^{1}$H NMR and (b) $^{13}$C NMR spectra of poly(OPA-*alt*-TDGA).

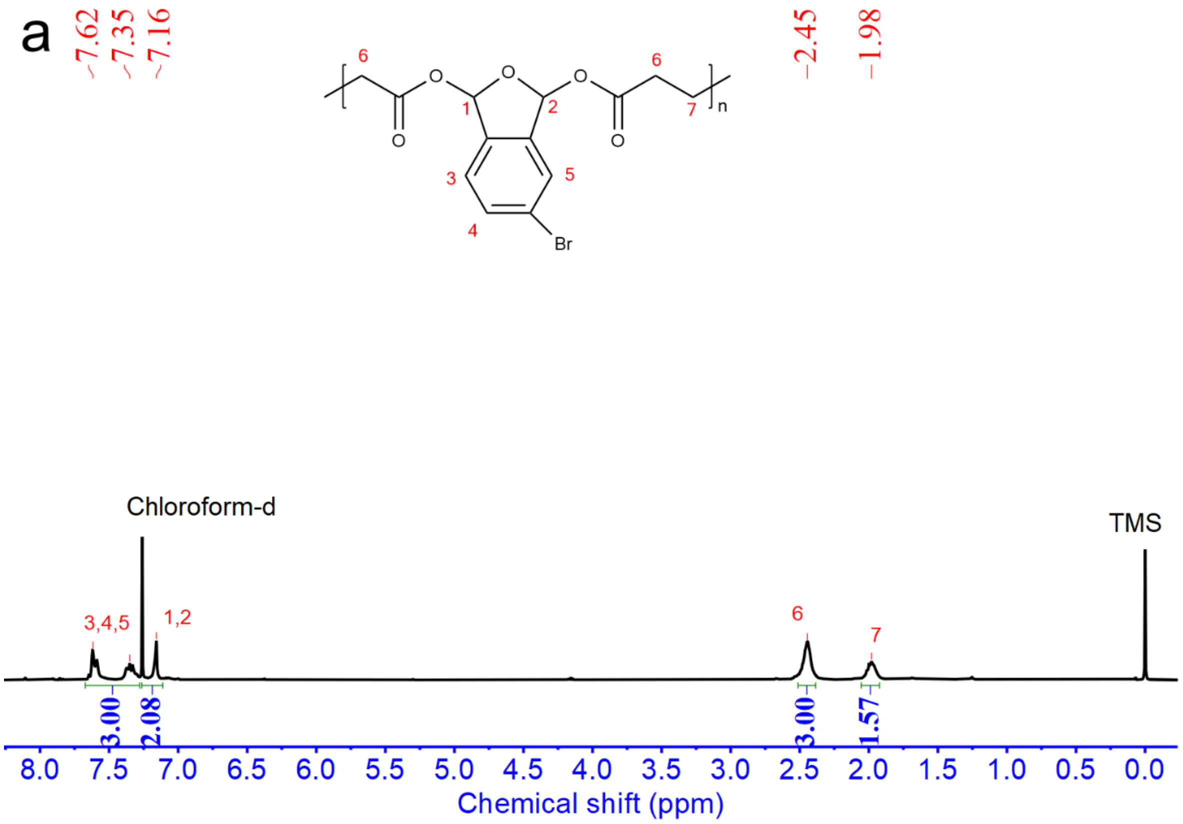


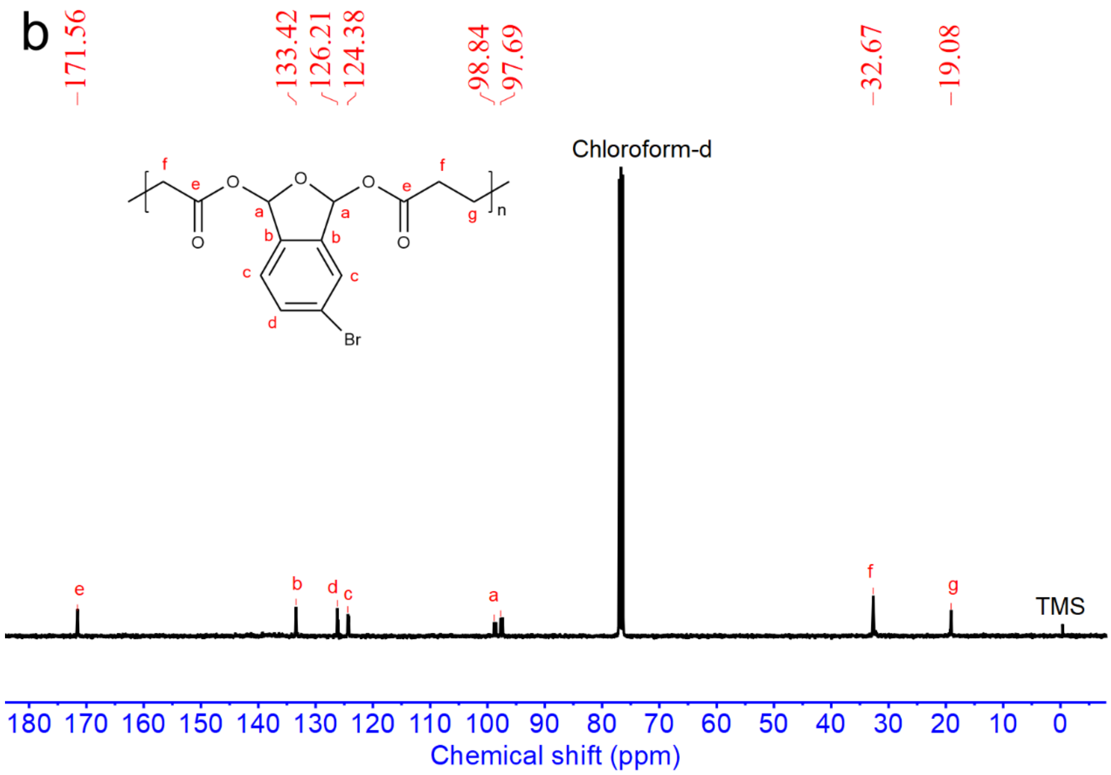


**Figure S15.** (a) $^{1}$H NMR and (b) $^{13}$C NMR spectra of poly(Br-OPA-*alt*-GA).

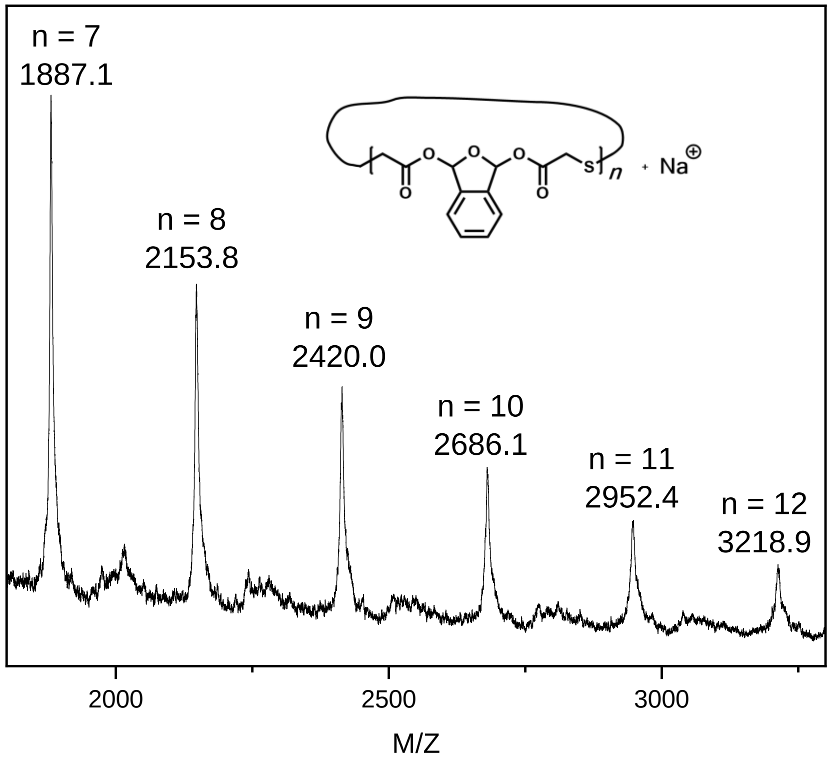


**Figure S16.** MALDI-TOF MS of cyclic poly(OPA-*alt*-TDGA).

**Table S1.** $T_g$ and $T_d$ values of the obtained polymers.

| | 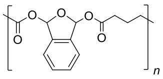 Poly(OPA-*alt*-GA) (13.2 kDa, 1.2) | 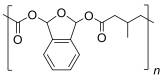 Poly(OPA-*alt*-MGA) (6.3 kDa, 1.4) | 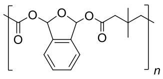 Poly(OPA-*alt*-DMGA) (4.5 kDa, 1.5) | 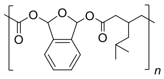 Poly(OPA-*alt*-*i*BuGA) (4.4 kDa, 1.2) | 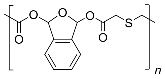 Poly(OPA-*alt*-TDGA) (6.3 kDa, 1.4) | 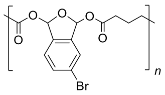 Poly(Br-OPA-*alt*-GA) (5.6 kDa, 1.2) |
|---|---|---|---|---|---|---|
| $T_g$ (°C) | 67 | 58 | 62 | 40 | 52 | 52 |
| $T_d$ (°C) | 200 | 190 | 177 | 183 | 203 | 185 |

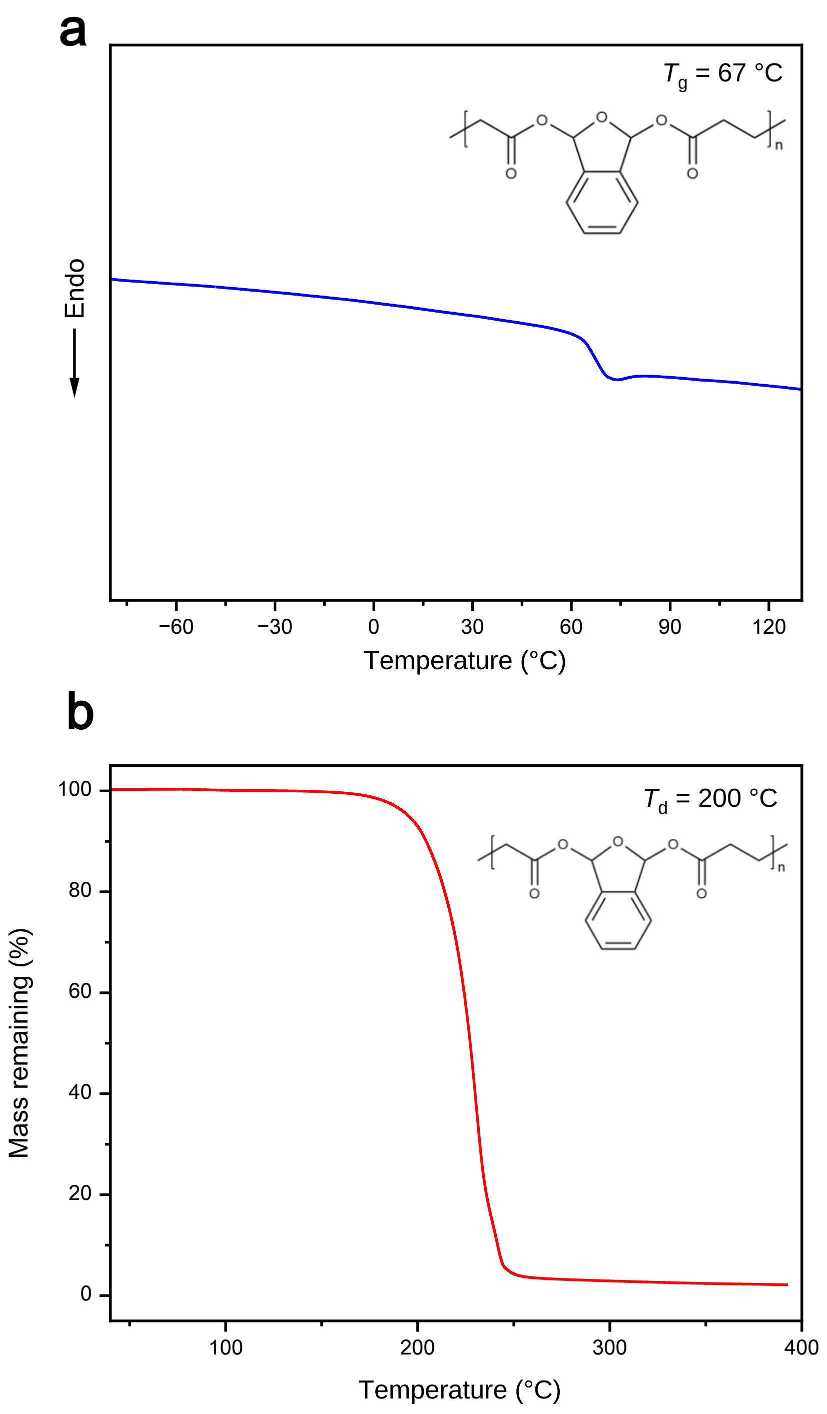


**Figure S17.** (a) DSC and (b) TGA curves of poly(OPA-*alt*-GA).

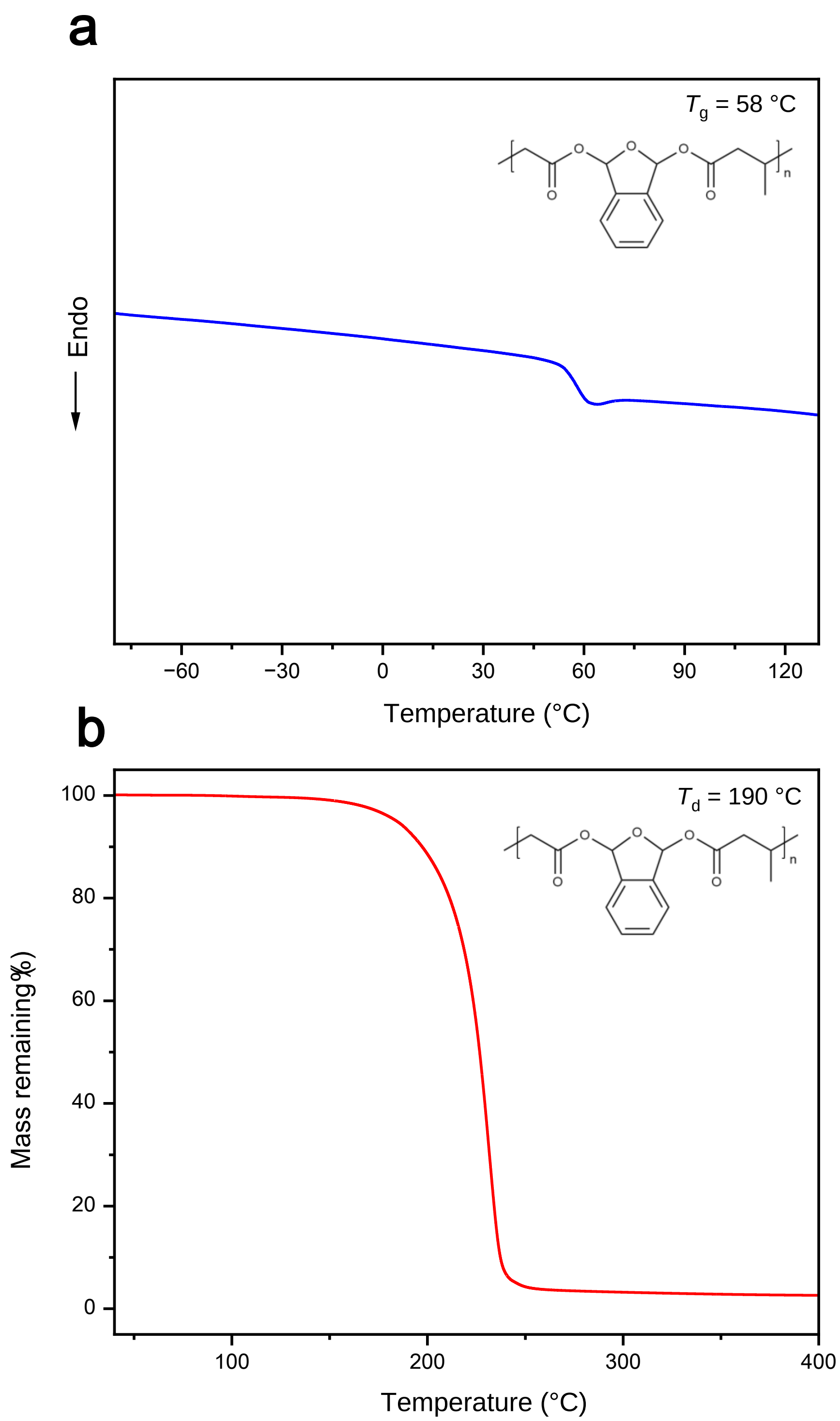


**Figure S18.** (a) DSC and (b) TGA curves of poly(OPA-*alt*-MGA).

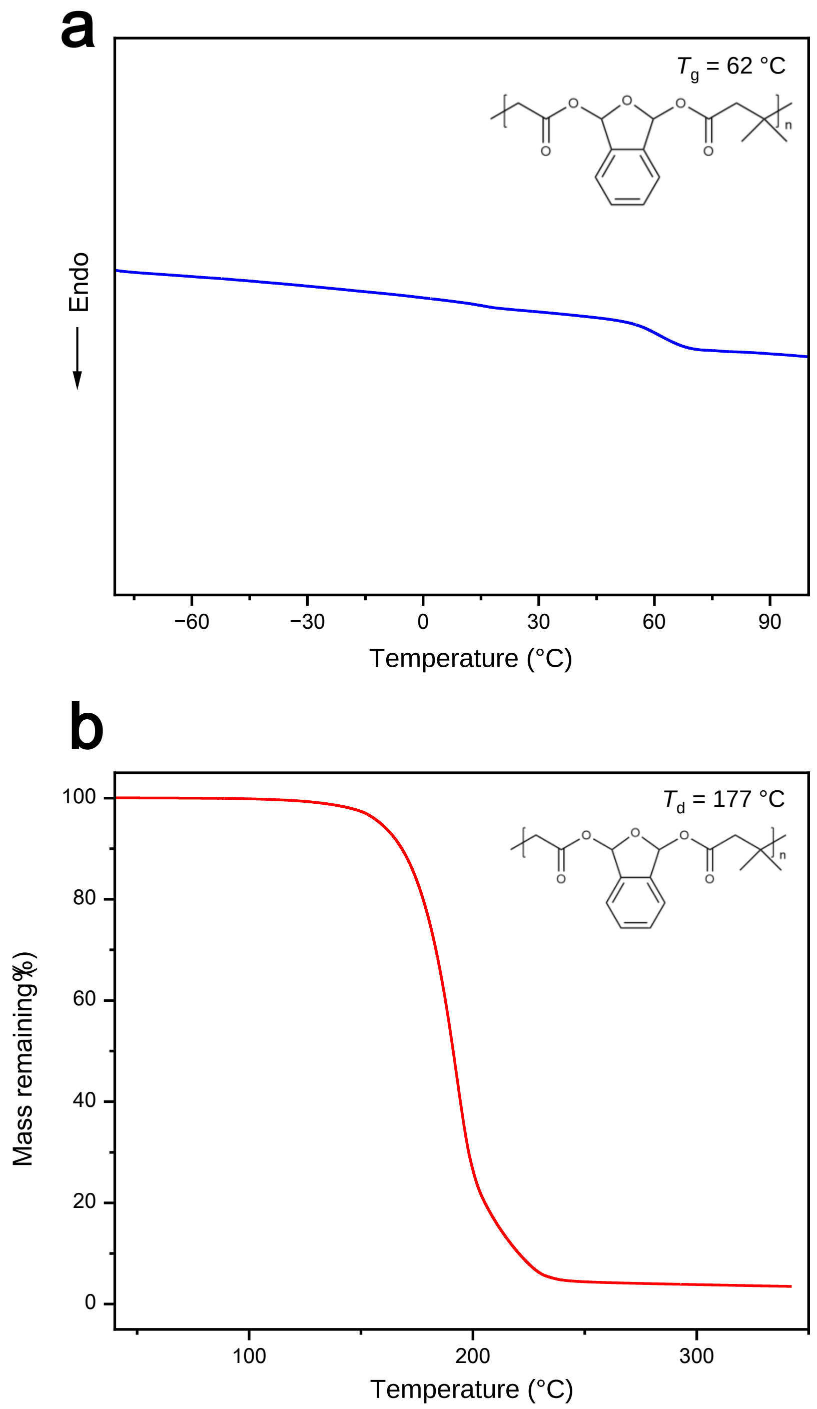


**Figure S19.** (a) DSC and (b) TGA curves of poly(OPA-*alt*-DMGA).

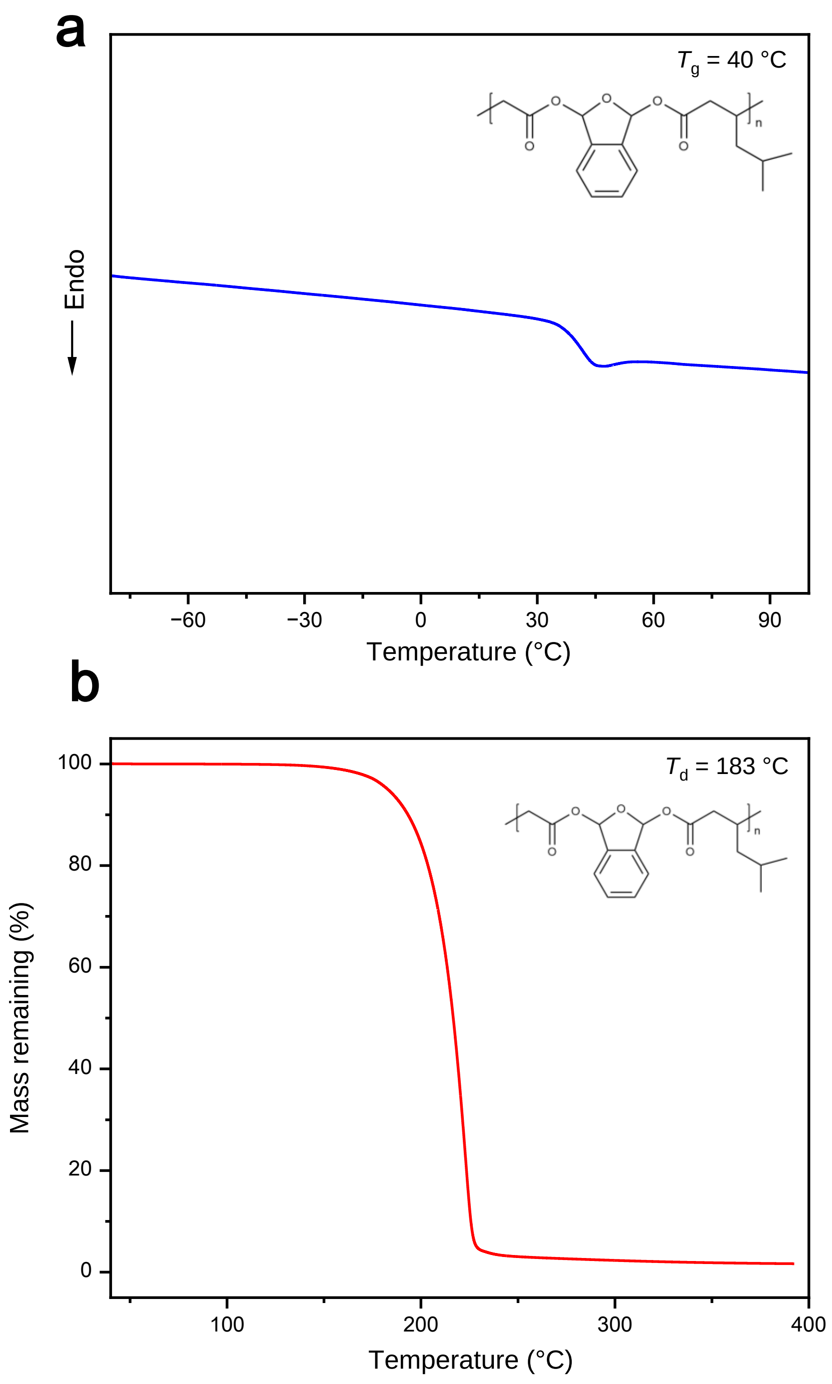


**Figure S20.** (a) DSC and (b) TGA curves of poly(OPA-*alt*-*i*BuGA).

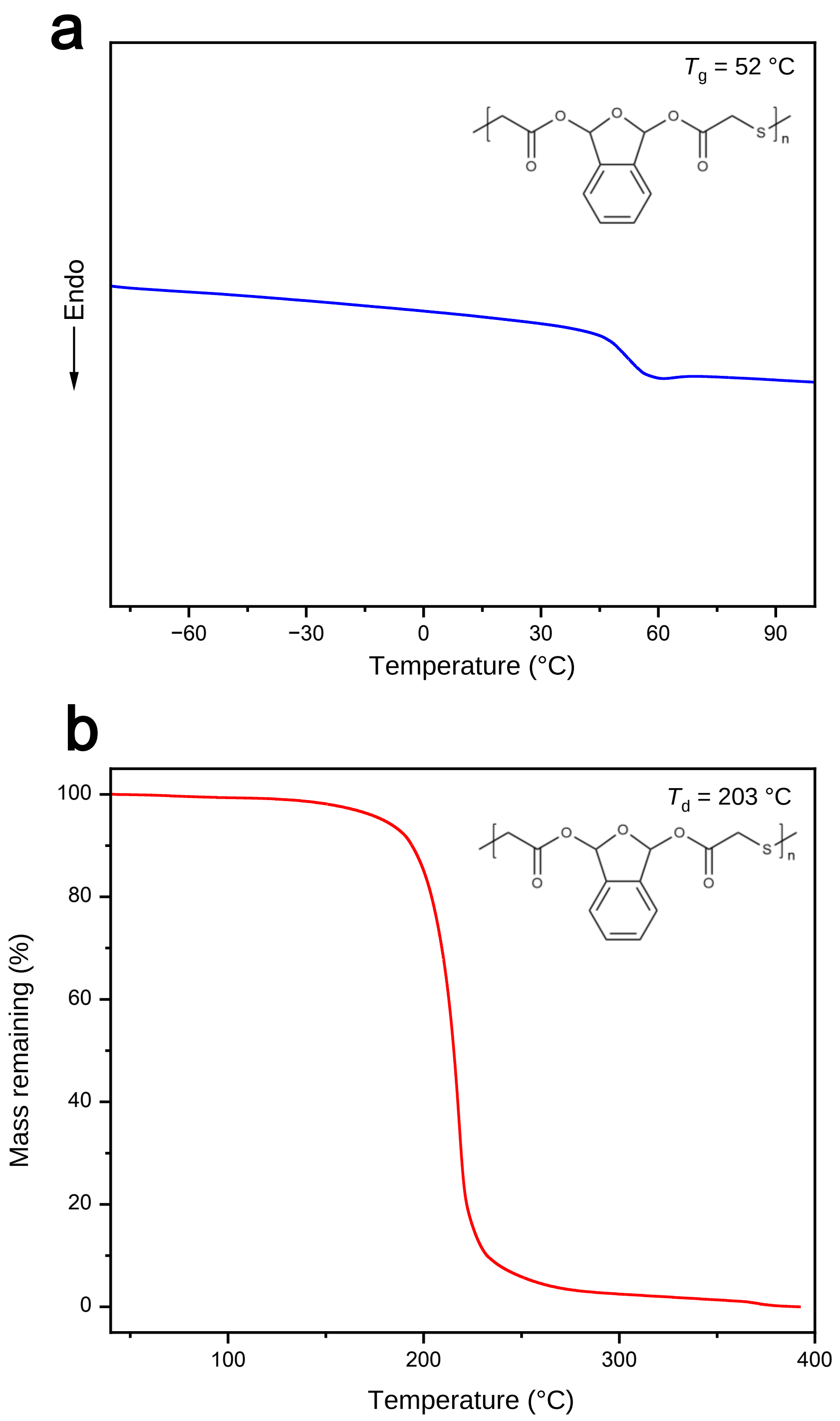


**Figure S21.** (a) DSC and (b) TGA curves of poly(OPA-*alt*-TDGA).

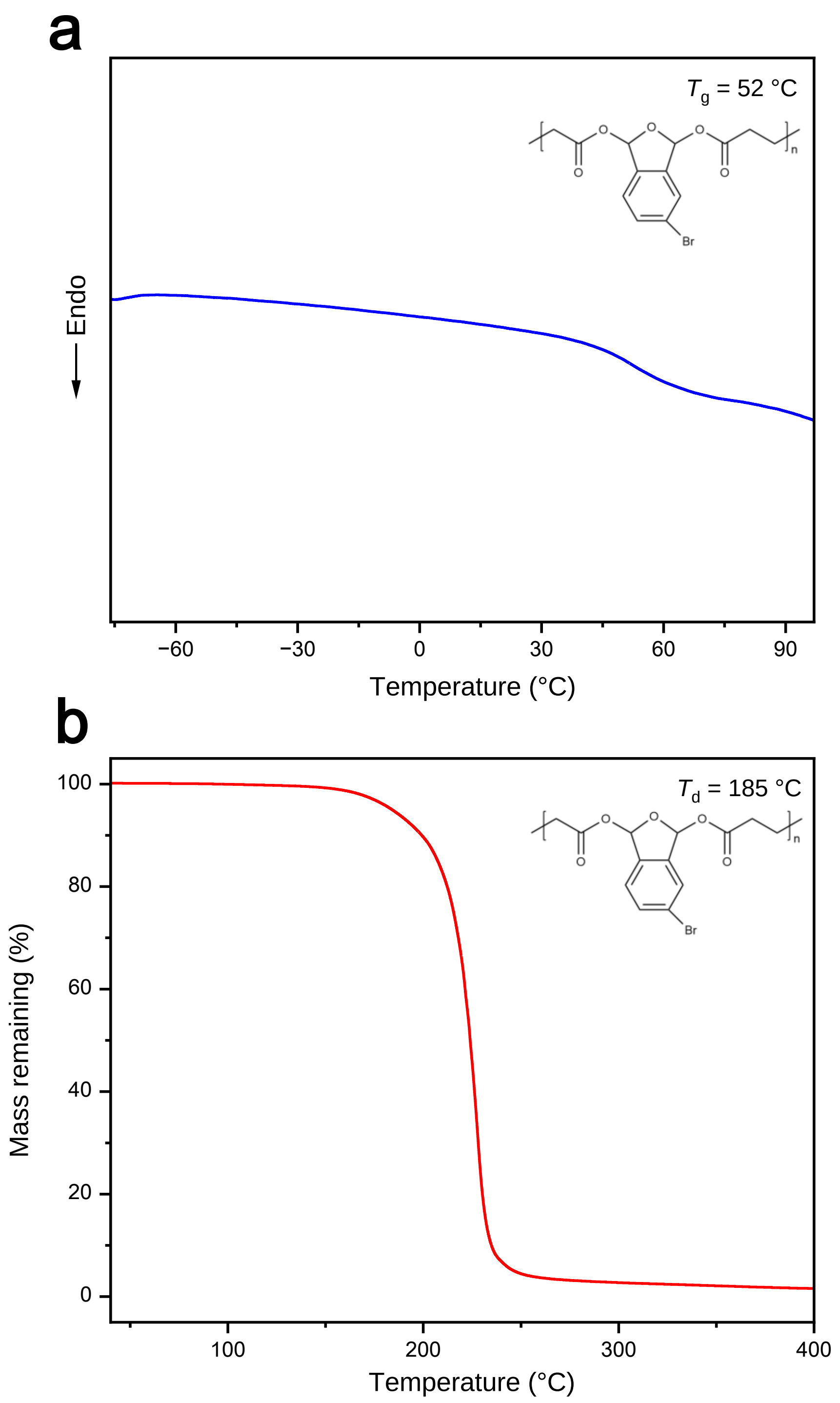


**Figure S22.** (a) DSC and (b) TGA curves of poly(Br-OPA-*alt*-GA).

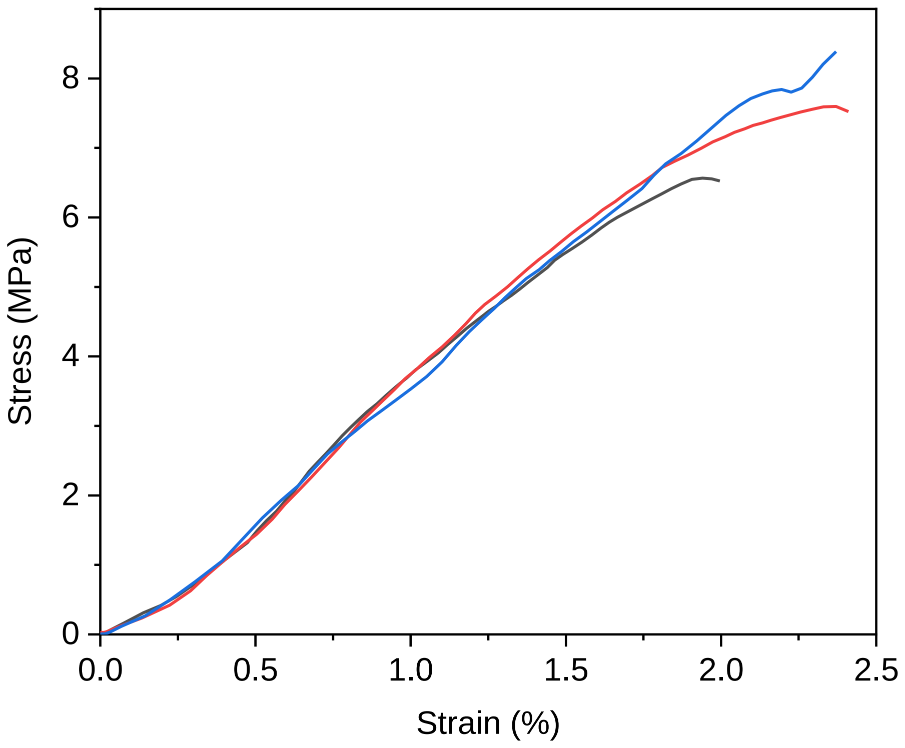


**Figure S23.** Stress-strain curves of the poly(OPA-*alt*-GA) (entry 9 in Table 1).

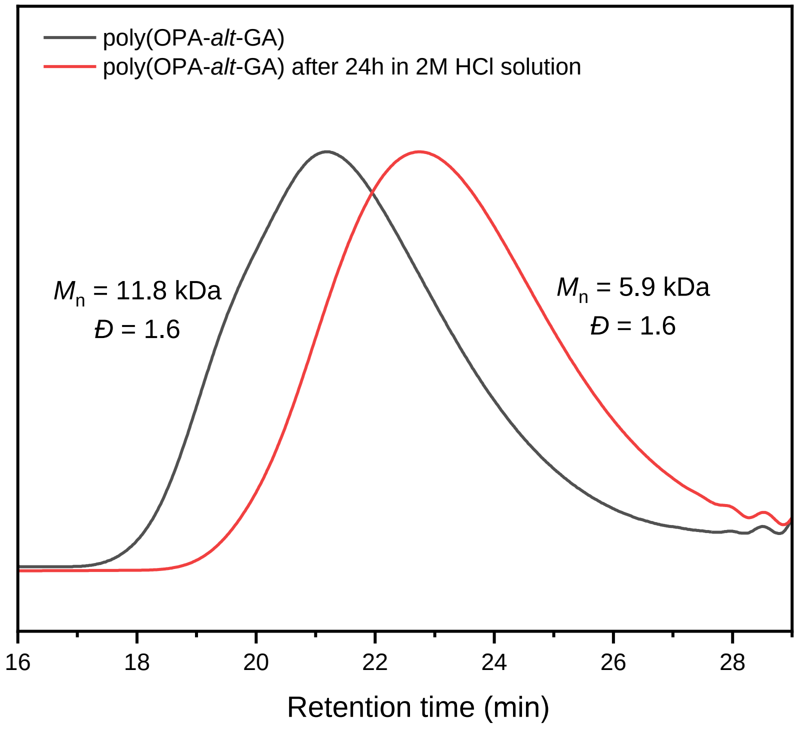


**Figure S24.** GPC curves of the poly(OPA-*alt*-GA) before and after stirred in 2 M HCl solution for 24 h.
